# Identification of Glutathione Peroxidase Gene Family in *Ricinus communis* and Functional Characterization of RcGPX4 in Cold Tolerance

**DOI:** 10.3389/fpls.2021.707127

**Published:** 2021-11-05

**Authors:** Xiaoyu Wang, Xuming Liu, Yong-qiang Charles An, Hongyu Zhang, Di Meng, Yanan Jin, Hongyan Huo, Lili Yu, Jixing Zhang

**Affiliations:** ^1^College of Life Science and Food Engineering, Inner Mongolia Minzu University, Tongliao, China; ^2^Horqin Plant Stress Biology Research Institute of Inner Mongolia Minzu University, Tongliao, China; ^3^U.S. Department of Agriculture-Agricultural Research Service, Plant Genetics Research Unit, Donald Danforth Plant Science Center, Saint Louis, MO, United States

**Keywords:** *Ricinus communis* L., genome-wide identification, *RcGPX4*, functional analysis, cold tolerance

## Abstract

Glutathione peroxidases (GPXs) protect cells against damage caused by reactive oxygen species (ROS) and play key roles in regulating many biological processes. Here, five *GPXs* were identified in the *Ricinus communis* genome. Phylogenetic analysis displayed that the GPXs were categorized into five groups. Conserved domain and gene structure analyses showed that the GPXs from different plant species harbored four highly similar motifs and conserved exon-intron arrangement patterns, indicating that their structure and function may have been conserved during evolution. Several abiotic stresses and hormone-responsive *cis*-acting elements existed in the promoters of the *RcGPXs*. The expression profiles indicated that the *RcGPXs* varied substantially, and some *RcGPXs* were coordinately regulated under abiotic stresses. Overexpression of *RcGPX4* in Arabidopsis enhanced cold tolerance at seed germination but reduced freezing tolerance at seedlings. The expression of abscisic acid (ABA) signaling genes (*AtABI4* and *AtABI5*), ABA catabolism genes (*AtCYP707A1* and *AtCYP707A2*), gibberellin acid (GA) catabolism gene (*AtGA2ox7*), and cytokinin (CTK)-inducible gene (*AtARR6*) was regulated in the seeds of transgenic lines under cold stress. Overexpression of *RcGPX4* can disturb the hydrogen peroxide (H_2_O_2_) homeostasis through the modulation of some antioxidant enzymes and compounds involved in the GSH-ascorbate cycle in transgenic plants. Additionally, RcGPX4 depended on the MAPK3-ICE1-C-repeat-binding factor (CBF)-COR signal transduction pathway and ABA-dependent pathway to negatively regulate the freezing tolerance of transgenic plants. This study provides valuable information for understanding the potential function of *RcGPXs* in regulating the abiotic stress responses of castor beans.

## Introduction

Plants are often exposed to diverse abiotic stresses, which significantly constrain the spatial distribution and yield of crops (Agarwal et al., 2006). These abiotic stresses can induce the accumulation of reactive oxygen species (ROS) (Choudhury et al., 2017). Although ROS can act as signaling molecules to activate the stress responses, high concentrations of ROS can damage various cellular components, leading to the death of cells (You and Chan, 2015). To survive, plants have developed antioxidant mechanisms for scavenging ROS, such as non-enzymatic and enzymatic antioxidants, to protect cells from uncontrolled oxidative damage (Blokhina et al., 2003). As a key ROS scavenger, glutathione peroxidases (GPXs, EC 1.11.1.9) belong to non-heme PODs and use reduced glutathione (GSH) or thioredoxin (Trx) as a reductant to convert organic hydroperoxides or hydrogen peroxide (H_2_O_2_) to the corresponding alcohols and water (Ursini et al., 1995; Bela et al., 2015; Passaia and Margispinheiro, 2015). Plant GPXs carry cysteine residue in the active site and have a preference for Trx as electron donors (Navrot et al., 2006; Koua et al., 2009).

A body of evidence has demonstrated that plant *GPXs* are responsive to abiotic stresses, such as drought, cold, salt, and oxidative stresses (Roxas et al., 2000; Yoshimura et al., 2004; Navrot et al., 2006; Passaia et al., 2013; Kim et al., 2014; Islam et al., 2015a). For instance, the transcript level of *Nelumbo nucifera NnGPX* was dramatically increased under cold, heat, salt treatments, and mechanical damage. Overexpression of *NnGPX* in rice has markedly improved salt tolerance (Diao et al., 2014). Overexpression of an endogenous GSH S-transferase with GPX activity in tobacco has enhanced seedling growth under cold stress compared with the control seedlings (Roxas et al., 2000). Two wheat *GPXs* were overexpressed in Arabidopsis and displayed higher salt and H_2_O_2_ tolerance compared to wild type (WT) (Zhai et al., 2013). *Rhodiola crenulata RcGPX5* conferred drought tolerance in transgenic plants by reducing the production of malondialdehyde (MDA) and increasing antioxidant enzyme activities (Zhang et al., 2019). Five *GPXs* were identified in rice and showed different responses to stress treatments (Passaia et al., 2013). In Arabidopsis, overexpression of chloroplastic *GPXs* increased tolerance to abiotic and biotic stresses. The reduced expression of both *GPX1* and *GPX7* resulted in severe morphological alterations in the leaves and chloroplasts of Arabidopsis (Chang et al., 2009). These findings revealed that GPXs regulated plant growth, development, and various abiotic stress responses.

To date, *GPX* genes from various plant species, such as *Panax ginseng* (Kim et al., 2014), *O. sativa* (Passaia et al., 2013), *Triticum aestivum* (Zhai et al., 2013), *Arabidopsis thaliana* (Miao et al., 2006; Chang et al., 2009; Gaber et al., 2012), *Populus trichocarpa* (Koh et al., 2007; Selles et al., 2012), and *Thellungiella salsuginea* (Gao et al., 2014), have been cloned and functionally characterized. Investigation of *GPX* families in *A. thaliana* (Gaber et al., 2012), *Lotus japonicus* (Ramos et al., 2009), *P. trichocarpa* (Navrot et al., 2006), *O. sativa* (Islam et al., 2015a), *Gossypium hirsutum* (Chen et al., 2017), and *Cucumis sativus* (Zhou et al., 2018) showed that the plant *GPX* family not only contains numerous *GPXs* with different subcellular localizations and physicochemical properties but also displays diverse abiotic stress responses. Thus, these studies emphasized the significance of *GPX* family studies on the genome-wide level.

*Ricinus communis* L. (*R. communis*) is an important non-edible oilseed crop, and its seed oil is widely used for pharmaceutical and industrial applications due to its high level of ricinoleic acid (Akpan et al., 2006; Ogunniyi, 2006; Scholza and da Silva, 2008). However, unfavorable environments, such as low temperature, severely inhibit the growth and development of the castor beans (Wang et al., 2019). Considering the increasing industry demands for castor bean oil, great effort is made to generate stress-tolerant and broadly adapted varieties by traditional breeding and genetic engineering techniques. It is imperative to reveal the molecular mechanism underlying the resistance of castor beans to inferior growth conditions. In this study, we identified *GPX* family genes in *R. communis* and analyzed their expression profiles under various stress conditions. Furthermore, *RcGPX4* was functionally characterized. Overexpression of *RcGPX4* in Arabidopsis decreased sensitivity to cold stress at seed germination but showed hypersensitivity to freezing treatment at seedlings compared with the WT. This study laid a foundation for developing new cold-tolerant castor cultivars.

## Materials and Methods

### Identification of *RcGPXs* and Protein Properties Analysis

To systematically identify the putative GPX in castor bean, the published GPX protein sequences, such as five in *O. sativa* (Islam et al., 2015a), eight in *A. thaliana* (Rodriguez Milla et al., 2003), six in *L. japonicus* (Ramos et al., 2009), six in *C. sativus* (Zhou et al., 2018), and 13 in *G. hirsutum* (Chen et al., 2017), were blasted against *R. communis* database.^1^ Furthermore, the GPX domain (PF00255)^2^ was used to search the *R. communis* database with HMMER software. The redundant sequences of GPXs were screened and removed. The remaining genes were confirmed by the existence of the GPX domain by Pfam. The molecular weights (kDa) and isoelectric points (PIs) were estimated using the ProtParam tool (Gasteiger et al., 2005). Subcellular localization was predicted by CELLO (Yu et al., 2006), WoLF PROST (Horton et al., 2007), and TargetP-2.0 (Armenteros et al., 2019).

### Analyses of Glutathione Peroxidases Genes/Proteins

Sequences of GPXs from rice, cucumber, Arabidopsis, and castor bean were aligned by Clustal W software (Larkin et al., 2007). The conserved motifs of GPXs were analyzed using the online MEME tool with the following parameters: the maximum number of motifs was set to 5 and the width of the motif was between 6 and 50 (Bailey et al., 2009). The exon-intron arrangement was analyzed by alignment of coding sequences (CDS) and genomic sequences using the online Gene Structure Display Server (GSDS) analysis tool (Hu et al., 2015). Seventy-five GPX protein sequences from *R. communis*, *C. sativus*, *O. sativa*, *A. thaliana*, *Vitis vinifera*, *T. salsuginea*, *Solanum lycopersicum*, *Phaseolus vulgaris Linn*, *P. trichocarpa*, *Phoenix dactylifera*, *Medicago sativa Linn*, *Citrullus lanatus*, and *Brassica napus* were used to perform comprehensive phylogenetic analysis using the Bayesian method. Briefly, a multiple sequence alignment of the selected amino acid sequences was automatically performed using Muscle (Edgar, 2004). The region used for generating the phylogenetic tree was obtained by excluding sites with alignment gaps (where gaps were found in over 5% of each alignment site) and missing data, leaving 166 positions in the final dataset. The best model of evolution was selected using Modelgenerator V.851 (Keane et al., 2006) following the corrected Akaike Information Criterion with four discrete gamma categories and used to construct the phylogenetic tree. The best model of evolution identified by the model generator was Le Gascuel (LG) + G (Le and Gascuel, 2008). But there were only 10 amino acid modes implemented excluding LG + G in MrBayes, the suboptimal available model Jones Taylor Thornton (JTT) + G (implemented under the name “Jones model” in MrBayes) was used (Jones et al., 1992). Finally, the phylogenetic analysis was inferred with MrBayes V.3.2.1 (Huelsenbeck et al., 2001) using the following parameters: 15 million generations, sampling every 1,000 generations, temperature = 0.2. The phylogenetic tree was decorated with Interactive Tree Of Life (iTOL) V5 (Letunic and Bork, 2021). The protein IDs and sequences of GPXs were listed in Supplementary Table 1. Total 1,500 bp sequences upstream from the translational start site of each gene were surveyed to predict *cis*-acting regulatory elements against the PlantCare database (Lescot et al., 2002).

### Plasmid Construction and Arabidopsis Transformation of *RcGPX4*

The CDS of *RcGPX4* was amplified from the castor bean leaves by real-time (RT)-PCR and inserted into the pCambia1300 expression vector under the control of cauliflower mosaic virus 35S promoter (CaMV 35S) with *Kpn*I and *Xba*I linkers using the primers GGGGTACCATGCTTTGTAGTTCTTCAACTAGAT, and GCTCTAGATCACGCAACCCCCAGCAGTTTCTTA. WT flowers of Arabidopsis (ecotype Columbia) were transformed with *Agrobacterium tumefaciens* GV3101 harboring CaMV35S:*RcGPX4* via the floral dip method (Clough and Bent, 1998). Transformants were selected in a media supplemented with 25 mg/L hygromycin and then confirmed by PCR. The homozygous T_3_ lines were used for further phenotypic investigation.

### Phenotypic Analysis of Transgenic Arabidopsis Plants

Seeds of transgenic plants and WT were sterilized with 5% NaClO and washed five times with distilled water. The sterilized seeds were stratified by dark incubation at 4°C for 3 days. For germination assay, the seeds were sown on 1/2 Murashige and Skoog (MS) and transferred into a growth chamber at 22°C as control, whereas grown at 4°C for cold treatment. Germinated seeds with protruded radicals were recorded for 13 consecutive days. The freezing tolerance assay was performed using 14-day-old seedlings, which were pre-treated at 4°C for 3 days, exposed to − 10°C for 5 h, thawed at 4°C for 12 h, and then recovered at 22°C for 5 days before counting survival rate.

### Measurement of Physiological Parameters Between Wild Type and Overexpressed Plants

Fourteen-day-old seedlings of overexpressed lines (OE) and WT grew on 1/2 MS were treated at 4°C for 0, 1, and 2 days for physiological indices assays. The content of H_2_O_2_, MDA, reduced GSH, oxidized GSH (GSSG), activities of L-ascorbate peroxidase (APX), superoxide dismutase (SOD), catalase (CAT), peroxidase (POD) was measured using reagent kits from Nanjing Jiancheng Bioengineering Institute of Jiangsu Province, China (Cat. nos. A064-1, A003-3, A061-1, A123-1-1, A001-1, A007-1-1, and A084-3-1). The activities of glutathione reductase (GR) and dehydroascorbate reductase (DHAR) were assayed by Suzhou Comin Biotechnology Co., Ltd., kits (Cat. nos. GR-2-W, DHAR-2-W).

### RNA Extraction and Expression Pattern Analysis

Three-week-old castor seedlings (genotype Tongbi5) planted in a growth chamber were subjected to different stresses, such as salt (300 mM), cold (4°C), and drought [15% Polyethylene glycol (PEG)] treatments. Total RNAs were extracted from leaves, stems, and roots at 0 and 8 h of stress treatments. The seeds of WT and OE lines imbibed for 1 day at 22°C and 8 days at 4°C were harvested and used for key genes expression involved in seed germination. The leaves of 4-week-old plants exposed to 4°C for 0, 30 min, 1 h, and 3 h were utilized for comparing the expression of cold stress-induced marker genes between WT and OE lines. Total RNA extraction, first-strand cDNA synthesis, and qRT-PCR were performed as described by Wang et al. (2019). The relative expression levels were calculated according to the 2^–ΔΔ^ ^Ct^ method (Livak and Schmittgen, 2001). *RcActin* (XM_002522148) and *RcGAPDH* (XM_002513282) were used as reference genes. The values are the means ± SD. The *P-*value < 0.05 of the Student’s *t*-test was considered to be statistically significant. The primers used in this study are presented in Supplementary Table 2.

## Results

### Identification of *RcGPXs* in the *Ricinus communis* Genome

Based on an extensive search in the *R. communis* genome database using the GPX domain and known GPXs of other plant species as Protein BLAST (BLASTp) queries, five GPXs were identified and designated as *RcGPX1* to *RcGPX5* according to the order of their gene locus (Table 1). The length of the RcGPXs was varied among members, ranging from 167 to 265 amino acids with an average of 201. The deduced molecular weights (MWs) and PIs of the five RcGPXs were 18.81–29.48 kDa and 4.97–9.20, respectively. Subcellular localization prediction indicated that RcGPX3 and RcGPX4 were localized in chloroplast/mitochondria, RcGPX2 and RcGPX5 were located in the cytoplasm, and RcGPX1 was located in cytoplasm/extracellular.

**TABLE 1:** Information on five *RcGPXs* identified in the *Ricinus communis* genome.

**Gene name**	**Locus name**	**Genomic position (5′-3′)**	**gDNA (bp)**	**ORF (bp)**	**Protein physicochemical characteristics**			
					**Length (aa)**	**MW (kDa)**	**pI**	**GRAVY**
*RcGPXl*	28153.m000283	28153:65967-68951	2,985	510	169	18.95	8.85	–0.418
*RcGPX2*	29657. m000470	29657:103446-106065	2,620	504	167	18.81	5.67	–0.416
*RcGPX3*	29848. m004526	29848:437789-439806	2,018	798	265	29.48	8.94	–0.154
*RcGPX4*	30190.m011204	30190:2501076-2503451	2,580	711	236	26.05	9.20	–0.270
*RcGPX5*	30190.m011205	30190:2504404-2507327	2,924	510	169	19.14	4.97	–0.396

*ORF; open reading frame, GRAVY; grand average of hydropathy.*

**FIGURE 1:**
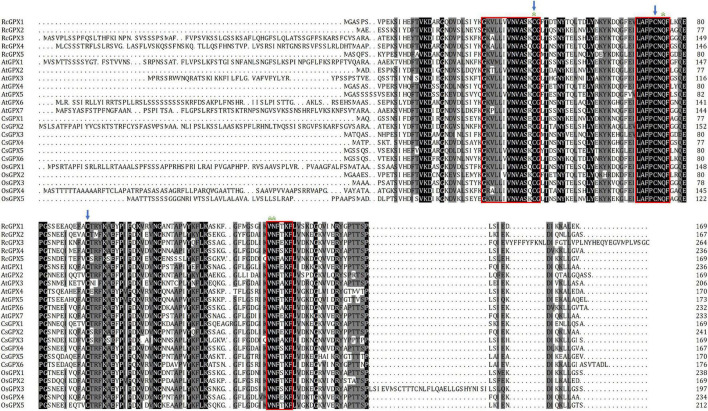
Multiple sequence alignments of the GPXs from the castor bean, cucumber, Arabidopsis, and rice. The three conserved domains found in most animal and plant GPXs were indicated with red boxes; the three conserved Cys residues of the plant GPX proteins were indicated with blue arrows. The amino acid residues that formed a potential redox center were indicated with asterisks. GPXs, glutathione peroxidases.

To analyze the properties of the five RcGPXs, the RcGPXs were aligned to identify the conserved residues within the families from the castor bean, cucumber, Arabidopsis, and rice using ClustalW. The results showed that three conserved Cys residues were identified in the members of the GPX family, and three conserved domains, GK(R)V(A/T/P)L(M)L(I)V(I)VNVASR(K/Q/E)CG, ILAFPCNQF and WNFT(E/S/A)KF were found in the GPXs of most plants. Several highly conserved residues, namely, Cys (C, known as a catalytic site), Gln (Q), Trp (W), and Asn (N), were identified in all of the aligned sequences, which might form a potential redox center (Figure 1).

### Gene Structures and Evolutionary Relationships Analyses of Glutathione Peroxidases in Castor Bean and Other Plant Species

**FIGURE 2:**
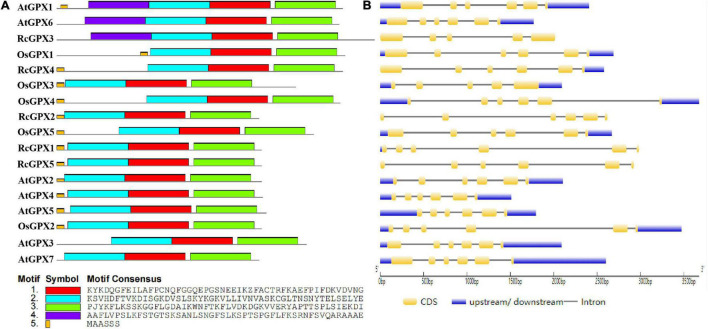
Conserved motifs and gene structures of GPXs from the castor bean, rice, and Arabidopsis. **(A)** Conserved motifs were identified by MEME, and their distribution was presented by different colors. **(B)** Exon/-intron organization was analyzed by GSDS software. The yellow round-corner rectangles indicated exons, the black lines indicated introns, and the blue rectangles indicated untranslated regions. GPXs, glutathione peroxidases; GSDS, Gene Structure Display Server.

To further investigate the evolutionary relationships among the GPXs, MEME analysis was used to identify the conserved motifs of GPXs from the castor bean, Arabidopsis, and rice. A total of five conserved motifs were found (Figure 2A and Supplementary Table 3). Motifs 1–4 were 50 amino acid residues, of which motifs 1–3 contained three conserved domains which are presented in Figure 1 separately, whereas motif 5 was 6 residues. Motifs 1 and 2 were associated with the GPX domain (PF00255) and existed in all tested GPX homologs. Motif 5, which was located in the N-terminal region, was present in all GPXs but absent in AtGPX3, AtGPX6-7, and RcGPX3. Interestingly, motif 4 was specific to AtGPX1, AtGPX6, and RcGPX3. Although some GPXs contained additional motifs, most interspecies GPXs shared similar motifs, which suggested that the structure and function of most GPXs may have been conserved in diverse plant species during evolution.

To determine gene structure diversity, alignments of genomic sequences against CDS were carried out by the online GSDS analysis tool (Figure 2B). All of the open reading frames (ORFs) of the five *RcGPXs* contained six exons and five introns except *RcGPX3*, which harbored five exons and four introns. Although the lengths of introns were variable, all *RcGPXs* showed the same lengths in exons 2–5 except *RcGPX3* that showed identical sizes in exons 2–4 with other *RcGPXs*. Notably, Some *GPXs* in the three species had a similar gene structure and comparable ORF lengths although they had variable lengths in 5′ or 3′ untranslated regions. Such features were also reported in *C. sativus*, *G. hirsutum*, and *T. salsuginea* (Gao et al., 2014; Chen et al., 2017; Zhou et al., 2018). These results revealed a significant degree of conservation of the *GPXs* family in the exon-intron arrangement in diverse plant species.

**FIGURE 3:**
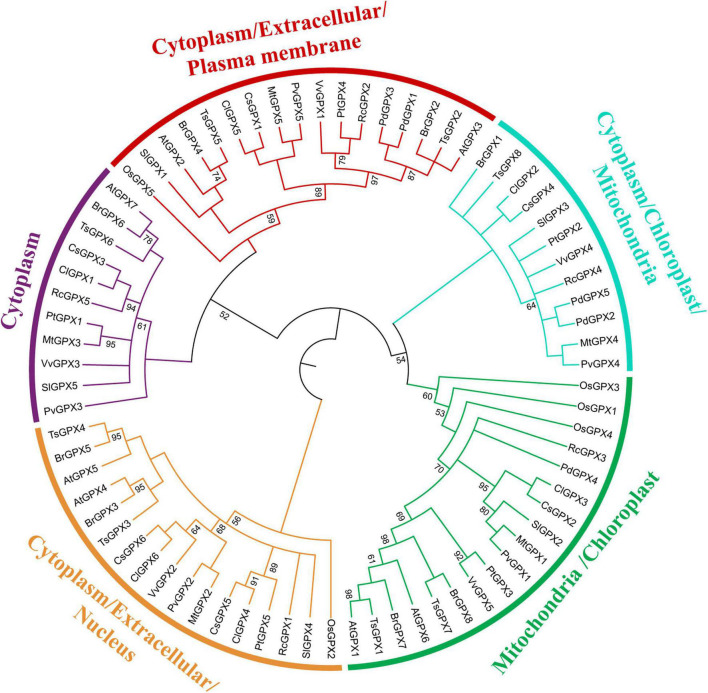
Phylogenetic tree of 75 GPX protein sequences based on 166 positions. The numbers at some branches indicated the posterior probability (posterior probability > 99 was not displayed). The tree topology as shown here was identical in the Bayesian inference (BI) analyses. The different colors of each branch indicated the different groups. GPXs, glutathione peroxidases.

The Bayesian method was constructed from thirteen 13 plant species, including such as 75 GPX homologs to explore their phylogenetic relationship (Figure 3). Combined with the prediction of CELLO, WoLF PSORT, and TargetP subcellular localization servers, plant GPXs were clustered into five groups: mitochondria and chloroplast localized GPXs, cytoplasm localized GPXs, cytoplasm/extracellular/plasma membrane localized GPXs, cytoplasm/mitochondria/chloroplast localized GPXs, and cytoplasm/extracellular/nucleus localized GPXs (Supplementary Table 4). RcGPXs were clustered into five phylogenetic groups and did not display a conserved clustering pattern along with the GPXs of diverse plant species. RcGPX1 was clustered within the subgroup of *S. lycopersicum* SlGPX4, RcGPX2 was clustered within the *P. trichocarpa* PtGPX4, RcGPX3 was categorized into the subgroup of *P. dactylifera* PdGPX4, RcGPX4 was classified along with PdGPX2/5, and RcGPX5 was clustered within the subgroup of ClGPX1 and CsGPX3.

### *Cis*-Acting Regulatory Elements Analysis of *RcGPX* Promoters

Numerous studies have revealed that GPXs are responsive to a variety of environmental stresses and play critical roles in protecting plants from environmental stresses (Miao et al., 2006; Gaber et al., 2012). Therefore, 1,500 bp sequences upstream from the translational start site of each gene were analyzed to identify *cis*-regulatory elements potentially important for differential transcriptional regulation of the *RcGPXs*. Diverse *cis*-acting regulatory elements were discovered and their quantity differed in each *RcGPX* promoter (Supplementary Table 5). Nearly all *RcGPX* promoters contained *cis*-acting elements responsive to abscisic acid (ABA) and drought and anaerobic stresses; some *RcGPX* promoters included elements responsive to salicylic acid (SA) and gibberellin acid (GA), whereas a few were related to low temperature, methyl jasmonate (MeJA), wound, auxin, and defense and stress responsiveness (Table 2). These findings suggested that the *RcGPX* genes might be associated with responses to different abiotic stresses and plant hormones.

**TABLE 2:** Putative *cis*-acting regulatory elements associated with stress and hormone responses in *RcGPX* promoters.

**Environmental stress or hormone**	***Cis*-acting regulator elements**	**Sequence**	***RcGPX***
Abscisic acid (ABA)	ABRE	ACGTG	1, 2, 4, 5
		TACGGTC	
		CACGTG	
		CGCACGTGTC	
Low-temperature responsiveness	LTR	CCGAAA	4
Drought inducibility	MBS	CAACTG	1, 2, 3, 4
Wound-responsive element	WUN-motif	AAATTTCCT	5
Anaerobic induction	ARE	AAACCA	1,3,4
MeJA responsiveness	CGTCA-motif	CGTCA	2
	TGACG-motif	TGACG	
Salicylic acid responsiveness	TCA-element	CCATCTTTTT	2, 5
Gibberellin-responsive element	P-Box	CCTTTTG	1, 2
	GARE-motif	TCTGTTG	
Auxin-responsive element	TGA-element	AACGAC	4
Defense and stress responsiveness	TC-rich repeats	GTTTTCTTAC	5

### Expression Analyses of the *RcGPXs* in Response to Various Abiotic Stresses

**FIGURE 4:**
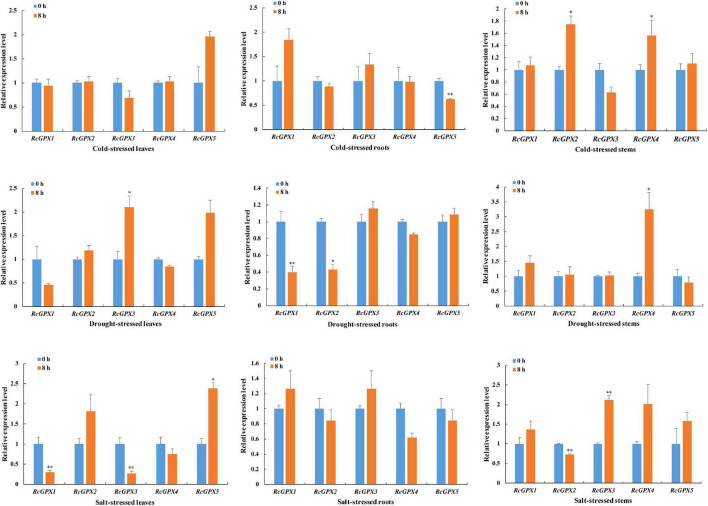
Effect of different stresses on the expression of the *RcGPX*s in leaves, roots, and stems. Total RNA was extracted from the leaves, stems, and roots of 3-week-old castor seedlings subjected to salt (300 mM), cold (4°C), and drought (15% PEG) treatments at 0 and 8 h. 0 h treated plants were used as controls. Data represent the means ± SD of three independent biological replicates. ^∗^*P* < 0.05, ^∗∗^*P* < 0.01 by Student’s *t*-test.

A growing body of evidence indicated that GPXs can protect the cells from damage caused by abiotic stresses (Roxas et al., 2000; Islam et al., 2015b; Zhang et al., 2019). Since stress-responsive *cis*-acting elements existed in the promoters of the *RcGPXs*, qRT-PCR was performed to investigate the expression change of *RcGPXs* under cold, drought, and salt stresses (Figure 4). Short-term exposure of *R. communis* plants to cold stress suppressed or induced the expression of some *RcGPXs*, and the effect differed among tissues, such as leaves, roots, and stems. Compared to the unstressed samples, the expression of all *RcGPXs* was not significantly changed under cold stress in leaves, while that of the only *RcGPX5* was significantly downregulated in roots, and *RcGPX2* and *RcGPX4* were dramatically upregulated in stems after cold treatment. The expression of *RcGPXs* was also regulated by salt and drought stresses. Salt stress (300 mM NaCl) highly increased the transcript level of *RcGPX5* and decreased the expression of *RcGPX1* and *RcGPX3* in leaves. In salt-treated stems, the expression of *RcGPX2* was downregulated and that of *RcGPX3* was upregulated. No obvious change in the abundance of all *RcGPXs* was observed in roots after salt stress. Drought stress induced the expression of *RcGPX3* and *RcGPX4* in leaves and stems, respectively. However, the transcript level of *RcGPX2* was decreased in roots after drought treatment.

### Overexpression of *RcGPX4* Enhanced Cold Tolerance at Seed Germination but Reduced Freezing Tolerance at Seedlings

**FIGURE 5:**
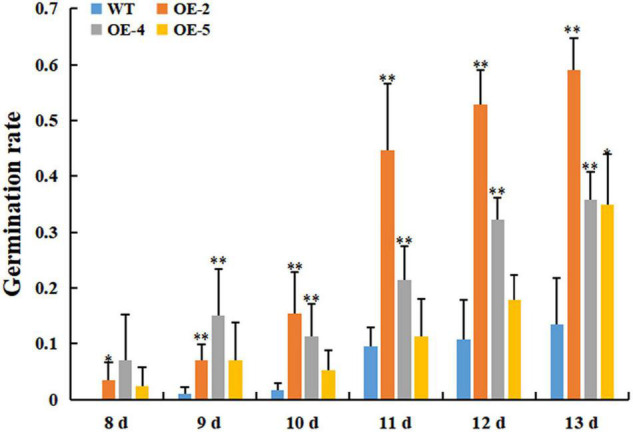
Overexpression of *RcGPX4* increased seed germination in transgenic plants under cold stress. The surface-sterilized seeds of wild type and transgenic plants were sown on 1/2 MS media and grown at 4°C for 13 consecutive days. Data represent the mean ± SD of three independent biological replicates (*n* = 42 seeds for each replicate). Asterisks indicate a significant difference between wild type and overexpressed lines (^∗^*P* < 0.05, ^∗∗^*P* < 0.01, student’s *t*-test). WT: wild-type; OE-2, OE-4, and OE-5: three T_3_ homozygous transgenic lines.

**FIGURE 6:**
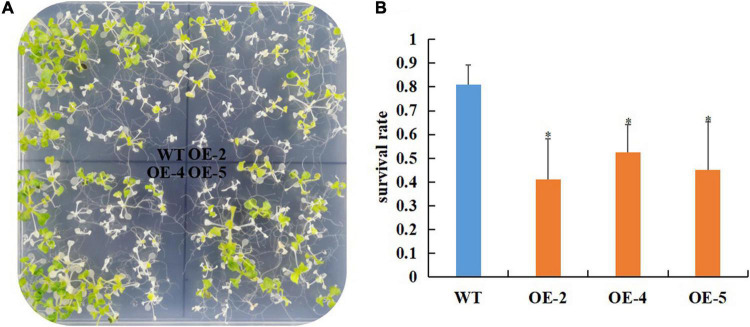
Overexpression of *RcGPX4* reduced freezing tolerance in transgenic plants. Freezing phenotype **(A)** and survival rate **(B)** of *RcGPX4* overexpressing transgenic plants. Fourteen-day-old seedlings grown 1/2 MS at 22°C were pre-treated at 4°C for 3 days, subjected to freezing stress at –10°C for 5 h, and then recovered at 22°C for 5 days before counting survival rate. Data represent the mean ± SD of three independent biological replicates (*n* = 42 seedlings for each replicate). Asterisks indicate a significant difference between wild type and overexpressed lines (^∗^*P* < 0.05, student’s *t*-test). WT: wild-type; OE-2, OE-4, and OE-5: three T_3_ homozygous transgenic lines.

Our previous study showed that the protein abundance of RcGPX4 was increased in cold-stressed imbibed seeds (Wang et al., 2019). To test whether *RcGPX4* might be associated with cold tolerance, three T_3_ homozygous transgenic lines were selected to investigate the effect on the phenotype. No difference in seed germination was observed between transgenic plants and WT under normal conditions (Supplementary Figure 1). When the seeds sown on 1/2 MS were exposed to cold stress, transgenic seeds began to germinate at day 7 whereas WT seeds were delayed 1 day. Moreover, the germination rate of OE lines was significantly higher than that of WT, which indicated that *RcGPX4* positively regulated seed germination of transgenic plants under cold stress (Figure 5). The freezing tolerance assay was done using 14-day-old seedlings. Surprisingly, after freezing stress, a higher survival rate was observed in WT compared with transgenic plants. The survival rate of WT was about 80% whereas that of OE lines was between 40 and 52%, which suggested that *RcGPX4* negatively mediated seedlings of transgenic plants in response to freezing treatment (Figure 6).

### Transgenic Plants Produced More H_2_O_2_ and Lipid Peroxidation Compared With Wild Type

**FIGURE 7:**
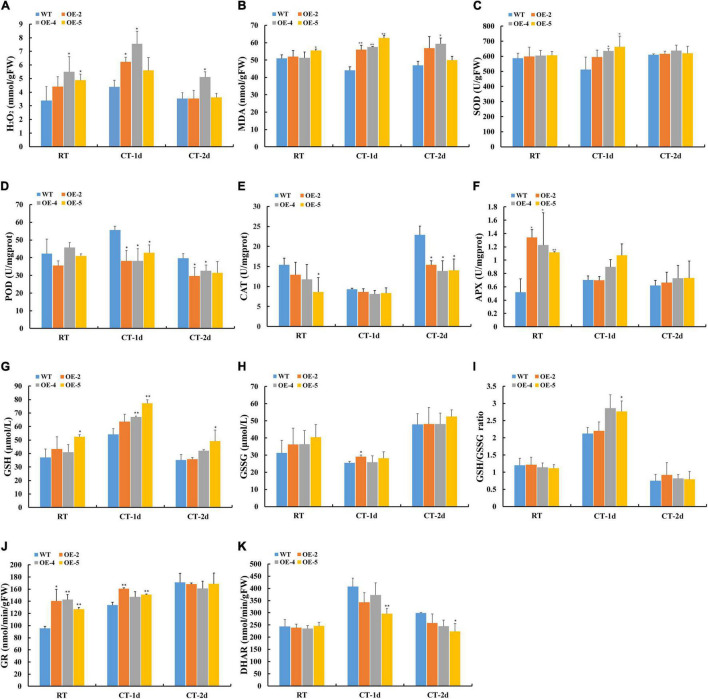
Effect of *RcGPX4* overexpression on metabolic content and the activities of antioxidant enzymes between WT and transgenic plants. **(A)** H_2_O_2_ content; **(B)** MDA content; **(C)** SOD activity; **(D)** POD activity; **(E)** CAT activity; **(F)** APX activity; **(G)** reduced GSH content; **(H)** GSSG content; **(I)** GSH/GSSG ratio; **(J)** GR activity; and **(K)** DHAR activity. RT: room temperature (22°C); CT-1: cold temperature (4°C) for 1 day; CT-2: cold temperature (4°C) for 2 days. FW: fresh weight. Data represent the mean ± SD of three independent biological replicates. Asterisks indicate a significant difference between wild type and overexpressed lines (^∗^*P* < 0.05, ^∗∗^*P* < 0.01, student’s *t*-test). WT, wild type; H_2_O_2_, hydrogen peroxide; MDA, malondialdehyde; SOD, superoxide dismutase; CAT, catalase; POD, peroxidase; GSSG, oxidized GSH; APX, activities of L-ascorbate peroxidase; GSH, glutathione; GR, glutathione reductase; DHAR, dehydroascorbate reductase.

Since GPX can act as an important detoxifier of H_2_O_2_, the amount of H_2_O_2_ was assayed between WT and transgenic lines. The content of H_2_O_2_ was significantly higher in transgenic lines than in WT under normal and cold stress conditions (Figure 7A). The alteration in the production of lipid hydroperoxide triggered by ROS can be determined by the level of MDA. The transgenic plants showed more accumulation of MDA compared with WT under cold stress, which suggested a higher degree of lipid peroxidation in transgenic plants (Figure 7B). Considering the surprising discovery that transgenic plants can accumulate more H_2_O_2_, we hypothesized that overexpression of *RcGPX4* might disturb the H_2_O_2_ homeostasis through the modulation of other antioxidant enzymes. In normal conditions, the activities of SOD and POD were not significantly different between WT and OE plants (Figures 7C,D). However, the activities of CAT and APX were obviously decreased and increased separately as compared to WT (Figures 7E,F). Under cold stress, the activity of SOD was significantly enhanced, the activities of POD and CAT were obviously decreased, while APX activity did not change noticeably in OE plants as compared to WT. To further investigate whether overexpression of *RcGPX4* can affect the antioxidant enzymes and compounds involved in the GSH-ascorbate cycle, we assayed the content of GSH and GSSG in WT and OE plants (Figures 7G,H). Under cold-stressed conditions, the content of reduced GSH and GSH/GSSG ratios was higher in transgenic plants than in WT (Figures 7G,I). The activity of GR significantly increased in OE lines in both conditions, while the activity of DHAR decreased after cold stress but did not display the obvious difference in the control condition (Figures 7J,K).

### Effect of *RcGPX4* Overexpression on the Expression of ABA/GA/CTK Pathway-Related Genes

**FIGURE 8:**
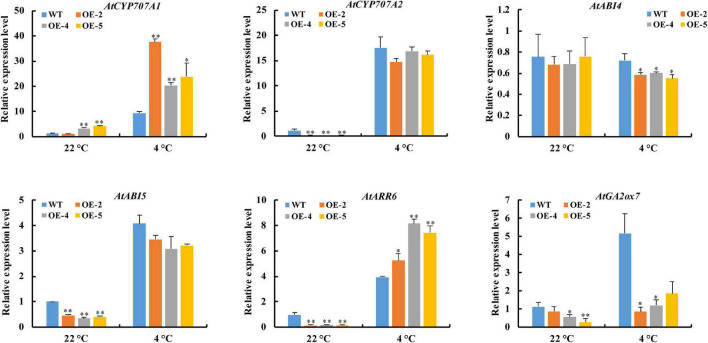
Expression pattern of ABA/GA/CTK pathway-related genes regulated by *RcGPX4*. The seeds of WT and transgenic lines imbibed for 1 day at 22°C and 8 days at 4°C were harvested for RNA extraction and qRT-PCR analysis. Data represent the mean ± SD of three independent biological replicates. Asterisks indicate a significant difference between wild type and overexpressed lines (^∗^*P* < 0.05, ^∗∗^*P* < 0.01, student’s *t*-test). ABA, abscisic acid*;* GA, gibberellin acid; CTK, cytokinin.

Since *RcGPX4* positively regulated the seed germination of transgenic lines under cold stress, we examined the expression of ABA/GA/CTK pathway-related genes in imbibed seeds of OE lines and WT under control condition and cold treatment. The results showed that the transcript of *AtABI4* in OE lines was significantly lower than in the WT under cold stress, whereas no obvious alteration was observed under the control condition. The expression level of *AtABI5* and *AtGA2ox7* was lower in OE lines compared with WT under both conditions. *AtCYP707A1* of OE lines showed a higher expression level than that of WT under both conditions. Compared with WT, a decrease in *AtCYP707A2* expression of OE lines was observed in both conditions, although it exhibited no significant difference in the presence of cold stress. Interestingly, the transcriptional expression of *AtARR6* in OE lines was decreased under control conditions but was increased under cold stress compared with WT (Figure 8). These results demonstrated that *RcGPX4* can confer cold tolerance of transgenic lines through the alteration of ABA/GA/CTK pathway-related genes under cold stress.

### Effect of *RcGPX4* Overexpression on the Expression of C-Repeat-Binding Factor-Dependent and ABA-Dependent Pathway Genes

**FIGURE 9:**
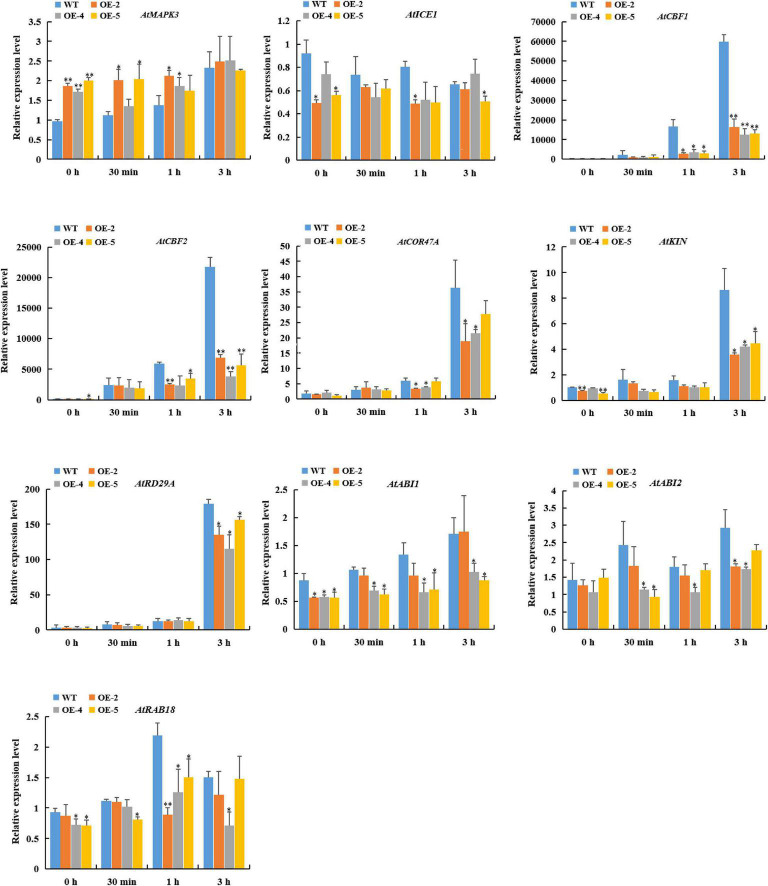
Expression pattern of cold-induced marker genes in WT and transgenic lines subjected to cold stress (4°C) for the indicated times. Data represent the mean ± SD of three independent biological replicates. Asterisks indicate a significant difference between wild type and overexpressed lines (^∗^*P* < 0.05, ^∗∗^*P* < 0.01, student’s *t*-test). WT, wild type.

To further explore the molecular mechanism of the reduced freezing tolerance regulated by *RcGPX4* in transgenic seedlings, we investigated the expression level of cold-responsive genes involved in CBF-dependent and ABA-dependent pathways. After cold treatment, the expression of *AtMAPK3* was significantly upregulated in OE lines compared with WT. Meanwhile, the expression of *AtICE1*, *AtCBF1*, *AtCBF2*, *AtCOR47A*, *AtKIN*, *AtRD29A*, *AtABI1*, *AtABI2*, and *AtRAB18* in transgenic plants was strikingly lower than in WT. These results suggested that *RcGPX4* played a negative role in freezing tolerance by suppressing the genes expression of the ICE1-CBF-COR cascade^3^ and ABA signaling pathways (Figure 9).

## Discussion

Unfavorable environmental stimulation can alter cellular redox homeostasis, which causes the excessive generation of ROS. Plants have evolved enzymatic and non-enzymatic antioxidants to neutralize excess ROS. GPXs, as efficient ROS scavengers, can defend plants against oxidative injury. Herein, a total of five *GPXs* were identified from the current *R. communis* genome. The number of *RcGPXs* was comparable to that in rice (five members), *L. japonicus* (six members), cucumber (six members), and *A. thaliana* (eight members). In our study, one tandem duplication event (*RcGPX4* and *RcGPX5*) was observed, which might be conducive to the expansion of the *RcGPX* gene family. The five RcGPXs harbored four highly similar motifs, which were also identified in all GPXs of other plant species (Zhou et al., 2018; Jana and Yaish, 2020). This indicates that the structure and function of GPXs from different plant species may have been conserved during evolution. A few conserved motifs were categorized into specific groups of GPXs, indicating that these motifs might be associated with the specific functions of the GPXs. Gene structure also showed that the GPXs from different species were highly conserved due to similar exon-intron organization and ORF lengths, which suggested that exon-intron arrangement played a critical role in the evolution of multigene families (Cao et al., 2016). The phylogenetic tree showed that GPXs from 13 plant species were classified into five groups based on predicted subcellular compartment, which was supported by the classification in other plants (Ozyigit et al., 2016). Margis et al. (2008) indicated that GPX clustered into the same subcellular compartments may be more associated with each other than GPXs with different localization from the same organism. Our results also showed that cytosolic localized GPXs were clustered together while chloroplast/mitochondria localized GPXs were categorized together. Ozyigit et al. (2016) suggested that the presence or absence or position of transit peptide sequences located in the N-terminal site of GPXs mainly determine the phylogenetic distribution. However, it will be needed to investigate whether GPXs, clustered into the same subcellular compartment branches, are positioned to predicted location and performed a similar function. Five RcGPXs were distributed into five phylogenetic groups, and each RcGPX was closer to GPX either in dicots or monocots, such as RcGPX3 and PdGPX4, RcGPX1 and SlGPX4, which indicated that GPX may not evolve separately in monocots and dicots. This observation was supported by phylogenetic analysis of *P. dactylifera* PdGPXs, which showed that PdGPX2, 4, 5 were highly related to homologs in *G. hirsutum* (Jana and Yaish, 2020).

A body of studies suggested that plant GPXs acted as crucial regulatory roles in response to diverse environmental factors. For example, the expression of rice *OsGPX2* and *OsGPX4* was upregulated after drought and oxidative stresses but downregulated under salt and cold stresses (Islam et al., 2015a). In cucumber, the transcript level of most *CsGPXs* was downregulated under salt and drought stresses (Zhou et al., 2018). Analysis of *RcGPX* promoter sequences showed that diverse *cis*-acting regulatory elements, such as environmental stress binding sites, were discovered and their quantity differed in each *RcGPX* promoter (Supplementary Table 5), which implied their potential roles in abiotic stresses. Therefore, we explored the responses of the *RcGPXs* to diverse stresses, such as cold, drought, and salt, in different tissues. The expression of *RcGPXs* varied largely under these stresses. At least one member of *RcGPXs* was significantly regulated in at least one tissue under specific environmental conditions except that all *RcGPXs* remained unaltered in cold-stressed leaves and salt-stressed roots. In addition, *RcGPX2* was upregulated in response to salt stress in leaves, whereas the transcript level for *RcGPX3* and *RcGPX5* was downregulated. This indicated that these members might act antagonistically to avoid a decrease in the reduced GSH or Trx pool.

Seed imbibition can rapidly generate H_2_O_2_ due to high respiratory activity and O_2_ depletion (Cakmak et al., 1993). Our proteomics study showed that the abundance of RcGPX4 was up-accumulated in the cold-stressed imbibed castor seeds compared to unstressed control, which might contribute to detoxify overproduction of H_2_O_2_ (Wang et al., 2019). This study displayed that overexpression of *RcGPX4* was insensitive to cold stress in the germination stage. It has been reported that H_2_O_2_ can interplay with the phytohormones, such as ABA and GA, to regulate abiotic stress responses in plants (Jubany-Mari et al., 2009; Ishibashi et al., 2012). ABA, GA, and CTKs function antagonistically to modulate seed germination and dormancy (Piskurewicz et al., 2008; Guan et al., 2014). AtABI4 acts as a positive regulator of primary seed dormancy, which can inhibit the transcripts of *AtCYP707A1* and *AtCYP707A2* to promote ABA biosynthesis while contributing to GA degradation via the upregulation of *AtGA2ox7* (Shkolnik-Inbar and Bar-Zvi, 2010; Shu et al., 2013, 2016). A recent study unraveled that AtABI4 can repress the transcription of type-A *ARRs*, one of CTK signaling components, and inhibit seed germination (Huang et al., 2017). In this study, the expression of *AtABI4* and *AtGA2ox7* was significantly downregulated while the transcription of *AtCYP707A1* and *AtARR6* was noticeably upregulated in cold-stressed imbibed OE seeds compared to unstressed control. Thus, we speculated that the improved seed germination of OE lines under cold stress was associated with the altered expression of ABA/GA/CTK pathway-related genes.

Cold stress can produce an increase in the amount of ROS, such as H_2_O_2_. The plant has evolved important physiological mechanisms, such as the GSH-ascorbate pathway, to cope with the damage caused by excessive H_2_O_2_. The accumulation and detoxification can be directly regulated by several enzymatic antioxidants. In our study, we surprisingly found that OE lines had higher H_2_O_2_ contents as compared to WT, which was not consistent with previous findings. After cold treatment, the transgenic plants had higher SOD activity but lower POD and CAT activities than WT. It was previously observed that transgenic plants overexpressed GPX had higher activities of SOD, CAT, and APX under drought and salinity stresses (Islam et al., 2015b; Zhang et al., 2019). APX activity was higher in transgenic seedlings than in WT under normal condition while was similar in both genotypes after cold stress. Roxas et al. (2000) showed that the elevated APX activity cannot substantially influence the general peroxide scavenging capacity. It seems to be a general phenomenon that overexpression of GPX can affect the activities of other antioxidant enzymes under abiotic stresses. However, based on our observation, the altered trend of antioxidant enzyme activities may depend on the function of different GPX family members. Therefore, an elevation of H_2_O_2_ in transgenic plants was mainly due to the decrease of CAT and POD activities and increase of SOD activity to cause the higher accumulation of MAD leading to lipid peroxidation and reduce tolerance to freezing stress. Moreover, we boldly speculated that maintenance of the high level of H_2_O_2_ regulated by the activities of different antioxidant enzymes may be needed as a signaling molecule to mediate the expression of cold-tolerant marker genes.

Most studies considered that plant GPX used Trxs as a reductant rather than GSH, which indicated that they were not relevant with GSH oxidation (Iqbal et al., 2006; Navrot et al., 2006). GSH can be oxidized to GSSG by DHAR, and GSSG can be recycled to GSH by GR (Rahantaniaina et al., 2017). Our results revealed that relatively high levels of GSH and GSH/GSSG in transgenic plants were controlled by the increased GR activity and the decreased DHAR activity, which might rebuild the new cellular redox balance in OE plants.

It is well-known that the ICE1-CBF-COR signaling pathway plays a critical role in plant cold acclimation. ICE1 can activate the transcription of *CBF* genes under cold stress, and CBF subsequently can induce the gene expression of *CORs* by directly binding their promoters, which contribute to plant freezing tolerance (Stockinger et al., 1997; Liu et al., 1998; Chinnusamy et al., 2003). However, this best characterized cold stress signaling pathway was regulated by diverse components, such as protein kinases, and phosphatases (Ding et al., 2019). Cold-activated mitogen-activated protein kinases MPK3 and MPK6 reduced ICE1 stability and transcriptional activity by phosphorylating ICE1 protein, which eventually decreased the *CBF* expression and plant freezing tolerance (Li et al., 2017). Besides the capacity to induce oxidative stress, H_2_O_2_ also can act as an important signaling molecule, which modulates the abiotic stresses tolerance by various pathways (Saxena et al., 2016). H_2_O_2_ can induce *MPK3* and *MPK6* via the activation of ANP1 (Kovtun et al., 2000). In this study, the expression level of *MPK3* was significantly higher in OE plants compared to WT in both conditions. However, the transcriptional level of *ICE1*, *CBFs*, and downstream *CORs* were lower in OE lines than in WT under cold stress. These results indicated that RcGPX4 depended on the MAPK3-ICE1-CBF-COR signal transduction pathway to negatively modulate freezing tolerance in transgenic plants. A previous study unraveled that ATGPX3 might regulate ABA and oxidative signaling by interacting with ABI1 and ABI2 (Miao et al., 2006). *ABI1/2* is involved in ABA signal transduction, and *RAB18* is the ABA-induced marker gene (Yao et al., 2020). Yao et al. (2020) showed that *CsbZIP18* reduced cold tolerance by suppressing the expression of *AtABI1/2* and *AtRAB18*. Our results found that the *AtABI1/2* and *AtRAB18* were downregulated in OE lines under normal conditions and cold stress, which suggested that *RcGPX4* impaired freezing tolerance in transgenic plants partly via the ABA-dependent pathway.

## Conclusion

In conclusion, a total of five *GPXs* were identified in castor beans through genome-wide analysis. Their physicochemical characteristics, subcellular prediction, and *cis*-acting elements were investigated. A comparison of phylogenetic relationships, protein motifs, and gene structures revealed that the structure and function of most GPXs may have been conserved in diverse plant species during evolution. The expression of the *RcGPXs* varied largely, and some *RcGPXs* were coordinately regulated under cold, drought, and salt stresses. Overexpression of *RcGPX4* in Arabidopsis decreased sensitivity to cold stress at the germination stage but showed hypersensitivity to freezing stress at seedlings. As compared to WT, transgenic plants can produce more H_2_O_2_ and lipid peroxidation, which was mainly due to the decrease of CAT and POD activities and the increase of SOD activity. The relatively high level of GSH and GSH/GSSG in transgenic plants was controlled by the increased GR activity and the decreased DHAR activity, which might rebuild the new cellular redox balance in OE plants. The improved seed germination in transgenic lines was associated with the alteration of ABA/GA/CTK pathway-related genes under cold stress. In addition, *RcGPX4* played a negative role in freezing tolerance by suppressing the genes expression of ICE1-CBF-COR cascade and ABA signaling pathways.

## Data Availability Statement

The original contributions presented in the study are included in the article/Supplementary Material, further inquiries can be directed to the corresponding authors.

## Author Contributions

XW and JZ conceived and designed the experiments. XW and XL performed the experiments. XW, XL, Y-qA, HZ, DM, YJ, and HH analyzed the data. XW wrote the manuscript. JZ, Y-qA, HZ, DM, and LY reviewed and edited the manuscript. All authors contributed to the article and approved the submitted version.

## Conflict of Interest

The authors declare that the research was conducted in the absence of any commercial or financial relationships that could be construed as a potential conflict of interest.

## Publisher’s Note

All claims expressed in this article are solely those of the authors and do not necessarily represent those of their affiliated organizations, or those of the publisher, the editors and the reviewers. Any product that may be evaluated in this article, or claim that may be made by its manufacturer, is not guaranteed or endorsed by the publisher.

## Footnotes

1
https://phytozome.jgi.doe.gov/pz/portal.html

2
http://pfam.xfam.org/

3 ICE1 = inducer of CBF expression 1; COR = cold-responsive.
