# Supplementary material for: Identification of Glutathione Peroxidase Gene Family in Ricinus communis and Functional Characterization of RcGPX4 in Cold Tolerance
Source: Front Plant Sci. 2021 Nov 5;12:707127. doi: 10.3389/fpls.2021.707127 (PMC8602854; doi:10.3389/fpls.2021.707127)

Supplementary Figure 1 Effect of *RcGPX4* overexpression on seed germination under normal condition. The surface-sterilized seeds of wild type and transgenic plants were sown on MS media and grown at 22 °C for three consecutive days. Data represent the mean±SD of three independent biological replicates (n=42 seeds for each replicate). WT: wild-type; OE-2, OE-4, OE-5: three T_3 homozygous transgenic lines.

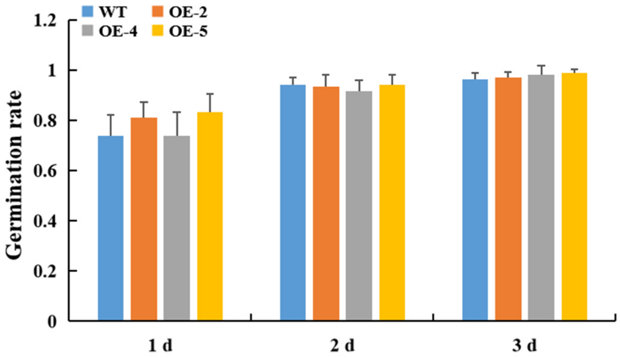

Supplement: Supplementary file 1 [file Image_1.pdf]
