# Supplementary material for: Identification of Glutathione Peroxidase Gene Family in Ricinus communis and Functional Characterization of RcGPX4 in Cold Tolerance
Source: Front Plant Sci. 2021 Nov 5;12:707127. doi: 10.3389/fpls.2021.707127 (PMC8602854; doi:10.3389/fpls.2021.707127)
Supplement: Supplementary file 2 [file Table_1.DOCX]

**Supplementary Table 1** GPX protein sequences used for phylogenetic tree analysis

| **Name** | **Protein ID** | **Species** |
| --- | --- | --- |
| RcGPX1 | 28153.m000283 | *Ricinus communis* |
| RcGPX2 | 29657.m000470 | *Ricinus communis* |
| RcGPX3 | 29848.m004526 | *Ricinus communis* |
| RcGPX4 | 30190.m011204 | *Ricinus communis* |
| RcGPX5 | 30190.m011205 | *Ricinus communis* |
| CsGPX1 | Csa4M013040.1 | *Cucumis sativus* |
| CsGPX2 | Csa4M651840.1 | *Cucumis sativus* |
| CsGPX3 | Csa5M154190.1 | *Cucumis sativus* |
| CsGPX4 | Csa5M154200.1 | *Cucumis sativus* |
| CsGPX5 | Csa6M408810.1 | *Cucumis sativus* |
| CsGPX6 | Csa7M392410.1 | *Cucumis sativus* |
| OsGPX1 | LOC_Os02g44500.1 | *Oryza sativa* |
| OsGPX2 | LOC_Os03g24380.1 | *Oryza sativa* |
| OsGPX3 | LOC_Os04g46960.1 | *Oryza sativa* |
| OsGPX4 | LOC_Os06g08670.1 | *Oryza sativa* |
| OsGPX5 | LOC_Os11g18170.1 | *Oryza sativa* |
| AtGPX1 | AT2G25080.1 | *Arabidopsis thaliana* |
| AtGPX2 | AT2G31570.1 | *Arabidopsis thaliana* |
| AtGPX3 | AT2G43350.1 | *Arabidopsis thaliana* |
| AtGPX4 | AT2G48150.1 | *Arabidopsis thaliana* |
| AtGPX5 | AT3G63080.1 | *Arabidopsis thaliana* |
| AtGPX6 | AT4G31870.1 | *Arabidopsis thaliana* |
| AtGPX7 | AT1G63460.1 | *Arabidopsis thaliana* |
| VvGPX1 | GSVIVG01010737001 | *Vitis vinifera* |
| VvGPX2 | GSVIVG01011101001 | *Vitis vinifera* |
| VvGPX3 | GSVIVG01019765001 | *Vitis vinifera* |
| VvGPX4 | GSVIVG01019766001 | *Vitis vinifera* |
| VvGPX5 | GSVIVG01035981001 | *Vitis vinifera* |
| TsGPX1 | Thhalv10000311m | *Thellungiella salsuginea* |
| TsGPX2 | Thhalv10001645m | *Thellungiella salsuginea* |
| TsGPX3 | Thhalv10001660m | *Thellungiella salsuginea* |
| TsGPX4 | Thhalv10006271m | *Thellungiella salsuginea* |
| TsGPX5 | Thhalv10017319m | *Thellungiella salsuginea* |
| TsGPX6 | Thhalv10023725m | *Thellungiella salsuginea* |
| TsGPX7 | Thhalv10026852m | *Thellungiella salsuginea* |
| TsGPX8 | Thhalv10028932m | *Thellungiella salsuginea* |
| SlGPX1 | Solyc06g073460.2.1 | *Solanum lycopersicum* |
| SlGPX2 | Solyc08g006720.2.1 | *Solanum lycopersicum* |
| SlGPX3 | Solyc08g080940.2.1 | *Solanum lycopersicum* |
| SlGPX4 | Solyc09g064850.2.1 | *Solanum lycopersicum* |
| SlGPX5 | Solyc12g056240.1.1 | *Solanum lycopersicum* |
| PvGPX1 | Phvul.001G041100.1 | *Phaseolus vulgaris Linn* |
| PvGPX2 | Phvul.001G149000.1 | *Phaseolus vulgaris Linn* |
| PvGPX3 | Phvul.002G157200.1 | *Phaseolus vulgaris Linn* |
| PvGPX4 | Phvul.002G288700.1 | *Phaseolus vulgaris Linn* |
| PvGPX5 | Phvul.002G322400.1 | *Phaseolus vulgaris Linn* |
| PtGPX1 | Potri.001G105100.1 | *Populus trichocarpa* |
| PtGPX2 | Potri.003G126100.1 | *Populus trichocarpa* |
| PtGPX3 | Potri.006G265400.1 | *Populus trichocarpa* |
| PtGPX4 | Potri.007G126600.1 | *Populus trichocarpa* |
| PtGPX5 | Potri.014G138800.1 | *Populus trichocarpa* |
| PdGPX1 | XP_008802381.1 | *Phoenix dactylifera* |
| PdGPX2 | XP_008794200.1 | *Phoenix dactylifera* |
| PdGPX3 | XP_008790648.1 | *Phoenix dactylifera* |
| PdGPX4 | XP_008790151.1 | *Phoenix dactylifera* |
| PdGPX5 | XP_008775339.1 | *Phoenix dactylifera* |
| MtGPX1 | Medtr1g014210.1 | *Medicago sativa Linn* |
| MtGPX2 | Medtr7g094600.1 | *Medicago sativa Linn* |
| MtGPX3 | Medtr8g098400.2 | *Medicago sativa Linn* |
| MtGPX4 | Medtr8g098410.1 | *Medicago sativa Linn* |
| MtGPX5 | Medtr8g105630.1 | *Medicago sativa Linn* |
| ClGPX1 | Cla011456 | *Citrullus lanatus* |
| ClGPX2 | Cla011457 | *Citrullus lanatus* |
| ClGPX3 | Cla021039 | *Citrullus lanatus* |
| ClGPX4 | Cla006080 | *Citrullus lanatus* |
| ClGPX5 | Cla010856 | *Citrullus lanatus* |
| ClGPX6 | Cla014745 | *Citrullus lanatus* |
| BrGPX1 | Brara.B02692.1 | *Brassica napus* |
| BrGPX2 | Brara.C02198.1 | *Brassica napus* |
| BrGPX3 | Brara.E00003.1 | *Brassica napus* |
| BrGPX4 | Brara.E01208.1 | *Brassica napus* |
| BrGPX5 | Brara.G01994.1 | *Brassica napus* |
| BrGPX6 | Brara.I01234.1 | *Brassica napus* |
| BrGPX7 | Brara.I04448.1 | *Brassica napus* |
| BrGPX8 | Brara.K00392.1 | *Brassica napus* |

>28153.m000283 RcGPX1

MGASPSVPEKSIHEFTVKDARGQDVDLSIYKGKVLLVVNVASKCGFTDTNYTQLTDLYNKYKDQGFEVLAFPCNQFLKQEPGSSEEAQEFACTRYKAEYPIFQKVRVNGANTAPVYKFLKASKFGFMGSGIKWNFTKFLVSKDGQVINRYGPTTSPLSIEDEIKKALEK

>29657.m000470 RcGPX2

MAEESSKSIYDFTVKDIRGNDVSLNEYSGKVLLIVNVASKCGLTQSNYKELNVLYEKYKNQGFEILAFPCNQFAGQEPGSNEEIQEVACTMFKAEFPIFDKIEVNGKNTAPLYKYLKSEKGGYFGDAIKWNFTKFLVNKEGKVVERYAPTTSPLKIEKDIQNLLGAS

>29848.m004526 RcGPX3

MASVPLSSPFQSLTHFKINPNSVSSSPSMAFFVPSVKSSLGSSKSAFLQHGFSLQLATSSGFFSKARSFCVSARAATEKSIHEYTVKDIDGKDVPLSKFKGKALLIVNVASKCGLTSSNYTELSHLYEKYKTQGFEILAFPCNQFGGQEPGSNPEIKNFACTRYKAEFPIFDKVDVNGPNTAPVYQFLKSSAGGFLGDLIKWNFEKFLVDKNGKVVERYPPTTSPFQIEVYFFFYFKNLDIFGTLVPLNYHEQYEGVMPLWSGCR

>30190.m011204 RcGPX4

MLCSSSTRFLSLRSVGLASFLVSKQSSFNSKQTLLQSFHNSTVPLVSRSINRTGNSRSVFSSLRLDHTMAAPSEPKSVHDFTVKDARGNDVDLSIYKGKALLIVNVASQCGLTNSNYTELTQLYQKYKDQGLEILAFPCNQFGSQEPGTNEQIMEFACTRFKAEYPIFDKVDVNGNNAAPIYKFLKSSKGGLFGDGIKWNFSKFLVDKDGNVVDRYAPTTSPLSIEKDVKKLLGVA

>30190.m011205 RcGPX5

MASQFSKYPESVHDFAVKDAKGNDVNLSIFKGKVLLIVNVASKCGMTNSNYTELNQLYDEYKDKGLEILAFPCNQFGDEEPGSNDEITEFVCSRFKSEFPIFDKIEVNGENSSSLYKFLKSGKWGIFGDDIQWNFAKFLVNKDGQVVDRYYPTTSPLSLEHDIKKLLGV

>Csa4M013040.1 CsGPX1

MAQGSSNSIFDFTVKDIRGNDVSLSEYKGKVLLIVNVASECGLTKSNYKELNVLYDKYKNQGFEILAFPCNQFAGQEPGNNEQIQETVCTRFKAEFPIFDKVDVNGKDAAPIYKFLKSQEAGRGLFGDGIKWNFTKFLVNKEGKVVGRYAPTTSPSKIEKDIENLLQSA

> Csa4M651840.1 CsGPX2

MSLSATFPAPIYVCSKTSTRFCYSFASWPSMAANLIPSLKSSLAASKSPFLRHNLTMQSSISRGVFSKARFSGVSARAATEKSIYDFTVKDIDGKGVSLNKFKGKVLLIVNVASRCGLTTANYSELSHLYEKYKAQGLEVLAFPCNQFGGQEPGSNPEIKQFACSRFKAEFPIFDKVDVNGPNTAPVYQFLKSSAGGFLGDLIKWNFEKFLVDKNGKVVERYPPTTSPFQIEKDIQKLVAA

> Csa5M154190.1 CsGPX3

MATQASNHPESIYDFTVKDAMGNDINLSIFKGKVLLIVNVASRCGMTNSNYVELNQLYEKYKEHGLEVLAFPCNQFGDEEPGSNDEIKDFVCSRFKSEFPIFDKAINGNNSAPLYKFLKLGKWGIFGDDIQWNFAKFLIDKNGNVVDRYYPPTPPLSIEHDIKKLLGIS

>Csa5M154200.1 CsGPX4

MATPSKTSVHDFTVKDAKGKDVDLSAYKGKVLLIVNVASQCGLTNSNYTELSQLYEKYKGHGFEILAFPCNQFGSQEPGSNEEIVQFACTRFKAEYPIFDKVDVNGNNAAPLYKFLKSSKGGLFGDAIKWNFSKFLVDKDGNVVDRYAPTTSPLSIEKDLKKLLGVA

>Csa6M408810.1 CsGPX5

MGSSQSVSEKSIHEFVVKDARGQDLDLSIYRGKVLLVVNVASKCGYTDSNYTQLTELYTKYKEKGLEILAFPCNQFLNQEPGSSQDAQEFACTRFKAEYPIFQKVNVNGPNTAPVYKFLKASKTGFLGTRIKWNFTKFLVDKEGHAIKRYGTTTTPLAIEADIKEALGEV

>Csa7M392410.1 CsGPX6

MGSSQSVTEKSIHEFTVKDFRGKDVNLNVYKGKVLLVVNVASKCGLTDSNYKQLTDLYNRYKDQDFEILAFPCNQFLKQEPGTSEDAQEFACTRYKAEYPIFQKVRVNGPDAVPVYKFLKATSNGFIGSRIKWNFTKFLIDKEGVVINRYGPTTNPLAIEVDIKKALGIASVTADL

>LOC_Os02g44500.1 OsGPX1

MPSRTAPFISRLRLLRTAAALSPFSSSAPPRHSPRILRAIPVGAPHPPRVSAAVSPLVRPVAAGFALFSMATAASSAASVHDFT

VKDASGKDVDLSTFKGKVLLIVNVASQCGLTNSNYTELSQLYEKYKDQGFEILAFPCNQFGGQEPGTNEEIVQFACTRFKAEYPIFDKVDVNGDNTAPIY

KFLKSSKGGLFGDNIKWNFSKFLVDKEGRVVERYAPTTSPLSMEKDIKKLLGSS

>LOC_Os03g24380.1 OsGPX2

MGAAESVPETSIHEFTVKDCNGKEVSLEMYKGKVLIVVNVASKCGFTETNYTQLTELYQKHRDKDFEILAFPCNQFLRQEPGSD

QQIKDFACTRFKAEYPVFQKVRVNGPDAAPLYKFLKASKPGLFGSRIKWNFTKFLIDKNGKVINRYSTATSPLSFEKDILKALED

>LOC_Os04g46960.1 OsGPX3

MAAAPSATSVHDFTVKDASGKDVNLSTYKGKVLLIVNVASQCGLTNSNYTELSQLYEKYKVQGFEILAFPCNQFGGQEPGSNEEIVQFACTRFKAEYPIFDKVDVNGNNAAPLYKYLKSNKGGLFGDSIKWNFSKFLVDKEGRVVDRYAPTTSPLSIEVMSCTTTCNLFLQAELLGSHYNISILSLQKDIKKLLGSS

>LOC_Os06g08670.1 OsGPX4

MASTTTTTAAAAARFTCLAPATRPASASASAGRFLLPARQWGAATTHGSAAVPVVAAPSRRWAPGVAYATAATGKSVHDFTVKD

IDGKDVALSKFKGRALLIVNVASQCGLTTANYTELSHLYEKYKTQGFEILAFPCNQFGAQEPGSNPQIKQFACTRFKAEFPIFDKVDVNGPNTAPIYKFL

KSSAGGFLGDLVKWNFEKFLVDKTGKVVERYPPTTSPFQIEKDIQKLLAA

>LOC_Os11g18170.1 OsGPX5

MAATTTSSSSGGGNRIWTSSLAVLALAVALVSLLSLRAPPPAAPSMADDLPTSVHDISVKDIKGNDVKLSEYEGKVLLIVNVAS

KCGLTNSNYKELNVLYEKYKEKGLEILAFPCNQFAGQEPGSNEEIEQTVCTRFKAEFPIFDKIDVNGKEAAPLYKFLKSQKGGFLGDGIKWNFTKFLVGKDGKVVERYAPTTSPLKIENDIQKLLGTS

>AT2G25080.1 AtGPX1

MVSMTTSSSSYGTFSTVVNSSRPNSSATFLVPSLKFSTGISNFANLSNGFSLKSPINPGFLFKSRPFTVQARAAAEKTVHDFTVKDIDGKDVALNKFKGKVMLIVNVASRCGLTSSNYSELSHLYEKYKTQGFEILAFPCNQFGFQEPGSNSEIKQFACTRFKAEFPIFDKVDVNGPSTAPIYEFLKSNAGGFLGGLIKWNFEKFLIDKKGKVVERYPPTTSPFQIEKDIQKLLAA

>AT2G31570.1 AtGPX2

MADESPKSIYDFTVKDIGGNDVSLDQYKGKTLLVVNVASKCGLTDANYKELNVLYEKYKEQGLEILAFPCNQFLGQEPGNNEEIQQTVCTRFKAEFPIFDKVDVNGKNTAPLYKYLKAEKGGLLIDAIKWNFTKFLVSPDGKVLQRYSPRTSPLQFEKDIQTALGQASS

>AT2G43350.1 AtGPX3

MPRSSRWVNQRATSKIKKFILFLGVAFVFYLYRYPSSPSTVEQSSTSIYNISVKDIEGKDVSLSKFTGKVLLIVNVASKCGLTHGNYKEMNILYAKYKTQGFEILAFPCNQFGSQEPGSNMEIKETVCNIFKAEFPIFDKIEVNGKNTCPLYNFLKEQKGGLFGDAIKWNFAKFLVDRQGNVVDRYAPTTSPLEIEKDIVKLLASA

>AT2G48150.1 AtGPX4

MGASASVPERSVHQFTVKDSSGKDLNMSIYQGKVLLIVNVASKCGFTETNYTQLTELYRKYKDQDFEILAFPCNQFLYQEPGTSQEAHEFACERFKAEYPVFQKVRVNGQNAAPIYKFLKASKPTFLGSRIKWNFTKFLVGKDGLVIDRYGTMVTPLSIEKDIKKALEDA

>AT3G63080.1 AtGPX5

MGASSSSSVSEKSIHQFTVKDSSGKEVDLSVYQGKVLLVVNVASKCGFTESNYTQLTELYRKYKDQGFVVLAFPCNQFLSQEPGTSEEAHQFACTRFKAEYPVFQKVRVNGQNAAPVYKFLKSKKPSFLGSRIKWNFTKFLVGKDGQVIDRYGTTVSPLSIQKDIEKALAQEL

>AT4G31870.1 AtGPX6

MAFSYASFSTPFNGFAANPSPITSAFLGPSLRFSTRTSKTRNPSNGVSVKSSNSHRFLVKSKNFSVYARAAAEKSVHDFTVKDIDGNDVSLDKFKGKPLLIVNVASRCGLTSSNYSELSQLYEKYKNQGFEILAFPCNQFGGQEPGSNPEIKQFACTRFKAEFPIFDKVDVNGPSTAPIYKFLKSNAGGFLGDIIKWNFEKFLVDKKGKVVERYPPTTSPFQIEKDIQKLLAA

>AT1G63460.1 AtGPX7

MATKEPESVYELSIEDAKGNNLALSQYKDKVLLIVNVASKCGMTNSNYTELNELYNRYKDKGLEILAFPCNQFGDEEPGTNDQITDFVCTRFKSEFPIFNKIEVNGENASPLYKFLKKGKWGIFGDDIQWNFAKFLVDKNGQAVQRYYPTTSPLTLEHDIKNLLNIS

>GSVIVG01010737001 VvGPX1

MAEAAPKSIYDFTVKDIRGNDVSLSDYNGKVLLIVNVASKCGLTHSNYKELNVLYEKYKSQGFEILAFPCNQFLGQEPGSNEEILEAACTMFKAEFPIFDKVEVNGKNTAPLYKFLKLQKGGLFGDGIKWNFTKFLVDKEGKVVDRYAPTTSPLKIEEDIQNLLGSA

>GSVIVG01011101001 VvGPX2

MGASQSGSEKSIHEFRVKDYKAKDVDLSVYKGKVLLVVNVASKCGLTDSNYTQLTELYNKYKDRGFEILAFPCNQFLKQEPGSSEQAQEFACTRYKAEYPIFHKVRVNGPDAAPVYKFLKAHKSGFLGSRIKWNFTKFLVDKEGTVLARYGPTTAPLTIEADIQKALGDK

>GSVIVG01019765001 VvGPX3

MSKQIQQGPESIYDFTVKDAEGKSVNLSIYKGKVLLIVNVASKCGLTNSNYTELNQLYEKYKDQGLEILAFPCNQFGEEEPGSNEQILEFVCTRFKSEFPVFDKIDVNGENAAPLYKFLKSGKWGIFGDDIQWNFGKFLVDKNGKIVDRYYPTTSPLTVENDVKKLLEVS

>GSVIVG01019766001 VvGPX4

MASQSSPQSVHSFTVKDARGNDVDLSIYKGKALLIVNVASQCGLTNSNYTELHQLYEKYKDQGLEILAFPCNQFGAQEPGSNEEIEKFVCTRFKAEYPIFDKIDVNGDSAAPLYKFLKSSKGGLFGDNIKWNFSKFLVDKDGKVVDRYAPTTSPLSIEKDIKKLLGIS

>GSVIVG01035981001 VvGPX5

MPSTKSSFGASNSAFLRTGFSLCSSEIPGVSLKSRFSGGVYARAATEKSLYDYTVKDIEKKDVPLSKFKGKVLLIVNVASKCGLTASNYSELSHIYEKYKTQGFEILAFPCNQFGGQEPGSNPEIKQFACTRFKAEFPIFDKVDVNGPTTAPVYQFLKSNAGGFLGDLIKWNFEKFLVDKNGKVVERYQPTTSPFQIEKDIQRLLAA

>Thhalv10000311m TsGPX1

massssyapfsaifngsrpnpsvkpaaflasslkfstvisnfanlsngfslkspinlgylfksryfnvqaraaaektvhdftvkdidgndvslnkfkgkvmlivnvasrcgltssnyselshlyekyksqgfeilafpcnqfggqepgsnpeikqfactrfkaefpifdkvdvngpstapiyeflksnaggflgglikwnfekflidkkg kvveryppttspfqiekdikkllaa

>Thhalv10001645m TsGPX2

mpksstwvsifflplafvfylyrypyspaivehssssiydisvkdiegndvslsqftgkvllivnvaskcglthgnykelnilyakykakgfeilafpcnqfgsqepgsneeikktvctmfkaefpvfdkievngenaaplykflkeqkgglfgdsikwnfakflvdkqgnvvdryapttspleiekdiekllasa

>Thhalv10001660m TsGPX3

mgasvsvpersvhqftvkdssgkelnlsiyqgkvllvvnvaskcgftesnytqltelyriykdhgfeilafpcnqflnqepgtsqeahefactrfqaeypvfqkvrvngqnaaplykflkaskpsflgsrikwnftkflvgkdglvidrygpmvtplsiekdikkaleea

>Thhalv10006271m TsGPX4

mgasisvseksihqftvkdssgkdvdlsvyqgkvllvvnvaskcgftetnytqltelyrkyrdqgfvilafpcnqfmyqepgtsqdahafactrfkaeypvfqkvrvngqnaapvykflkskkptflgtrikwnftkflvgkdgqvidrygptvpplsiendikkalgelpsvnp

>Thhalv10017319m TsGPX5

madespksiydftvkdiggndvslsqfkgktllivnvaskcgltdanykelnvlydkykeqgleilafpcnqflgqepgnneeiqqtvctkfkaefpifdkvdvngkntaplykylkaekggllidaikwnftkflvspdgkvfqrysprtsplqfendiqtllgqasss

>Thhalv10023725m TsGPX6

matkgpesvyeitiedakgdsfelsqykgkvllivnvaskcgmtnsnytelnelynkykdkgleilafpcnqfgeeepgttdqitdfvctrfksefpifnkievngenasplykflkkgkwgifgddiqwnfakflvdktgqaveryypttspltlehdikkllnis

>Thhalv10026852m TsGPX7

massyapfsavfngfaatkpnpprnssaflapsknfstetsnsrnlrngvslksssnhgfrfksrnfsvyaraasektvhdftvkdingkdvslskfkgkpllivnvaskcgltssnyselshlyekyknqgfeilafpcnqfggqepesnpdikrfvctrfkaefpifdkvdvngpstapiyqflksksggflgefikwnfekflvdkngkvveryppt tspfqiekdi qkllaa

>Thhalv10028932m TsGPX8

mlrssfrllyirtnhlvrvsssssslslslfpskfdsakplfnshrirplplsttgaklsrsehsmaatsepksvydftvkdakgndvdlstykgkvllivnvasqcgltnsnytelaqlyqkykdhgfeilafpcnqfgnqepgsneeivqfactrfkaeypifdkvdvngdkaapiykflksskgglfgdgikwnfakflvdkdgkvv dryapttsplsiekdlkkmlgvta

>Solyc06g073460.2.1 SlGPX1

MAEGSPKSIYDFTVKDIQGNEVPLSNYRGKVLLIVNVASKCGLTDSNYKELNILYEKYKDQGFEILAFPCNQFLWQEPGTNEEIQQTVCTRFKAEFPVFEKIDVNGDNAAPLYKFLKSEKGGFLGSAVKWNFTKFLVDKEGKVVERYAPKTPPLQFEKDIKNLLGVA

>Solyc08g006720.2.1 SlGPX2

MASFASSSTFSPICTFFSKPKLNLYCNSSSSSSYKPTCNSSKSSFFQNGFSLLTAKSFGSSLKSKNMANTIYARAATEKTIYDFTVKDIDGKDVPLSTFKGKVLLIVNVASRCGLTTSNYTELSSVYEKYKNQGLEILAFPCNQFGAQEPGSNPEIKQFACTRFKAEFPIFDKVDVNGPNTAPVYQFLKSSAGGFLGDLVKWNFEKFLVDKNGKVVERYPPTTSPLQIEKDIQKLLAA

>Solyc08g080940.2.1 SlGPX3

MLCSTARVLLIPKQNLSILRRFSSILRQTQFNSVFNLPVSSFSKPIRTVLPNSLVTSKGFELWGLRSDHTMASQSSNPQSVYDFTVKDAKGKDVDLSIYKGKVLIIVNVASQCGLTNSNYTDMTELYKKYKDQGLEILAFPCNQFGGQEPGNIEDIQQMVCTRFKAEYPIFDKVDVNGDNAAPLYRFLKSSKGGFFGDGIKWNFSKFLIDKEGHVVDRYSPTTSPASMEKDIKKLLGVA

>Solyc09g064850.2.1 SlGPX4

MGASKSVPQKSIYEFTVKDSKGKNVDLSIYKGKVLLVVNVASKCGFTSTNYTQLTQLYNEYKDKDFEVLAFPCNQFLKQEPGTSEQAQEFACTRFSAEYPIFQKVRVNGPNEAPVYTFLKASKGGFLSRSIKWNFTKFLVDKEGKVIRRYGSTTPPLSIKGDIEKALGEN

>Solyc12g056240.1.1 SlGPX5

MAGQPEKKPQSVYDFSLKDATGNDVDLSIFKGKVLLIVNVASKCGMTNSNYTELNQLYEKYKDQGLEILAFPCNQFGEEEPGTNDQILNFVCTRFKSDFPIFDKIEVNGENASPLYKFLKSGKWGIFGDDIQWNFAKFLVDKNGQVVDRYYPTTSPLTIERDMKKLLETI

>Phvul.001G041100.1 PvGPX1

MRHNLSSIFQLKKGSNLRTLFTSQSPKMSSMAFSTTFFTSLHDFTHTRTNPPNSPSFPFIKSSLASSNSTFFHPTLSLQTLSTFPRFIKPRSFSVHARAATEKTIYDFTVKDIDRKDVPLNKFKGKVLLIVNVASRCGLTSSNYSELSRLYEKYKNQGLEVLAFPCNQFGMQEPGSNEEIKQFACTRYKAEFPIFDKVDVNGPFTAPVYQFLKSSAGGFLGDLIKWNFEKFLVDKNGKVIERYPPTTSPFQIEKDIQRLLAA

>Phvul.001G149000.1 PvGPX2

MGASESVSEKSIHEFTVKDAKGRDVNLTTYKGKVLLVVNVASKCGFTDSNYTQLTELYGRCKDKGFEILAFPCNQFLKQEPGNSLEAEQFACTRYKAEYPIFRKVRVNGPDTAPVYKFLKANKPGCMGSRIKWNFTKFLVDKEGNVLKRYGPTTSPLSIEKDIKMALG

>Phvul.002G157200.1 PvGPX3

MTTVSSNDSKSVYDFSVKDAKGDVVDLSTYEGKVLLIINVASKCGLTNSNYTELNDLYAKYKEEGLEILAFPCNQFGEQEPESNDKIVDFVCTRFKSEFPIFDKIEVNGGNSAPLYKFLKSGKWGIFGDDIQWNFAKFVVDKKGQVVGRYYPTTSPLSLERDIRQLLGIS

>Phvul.002G288700.1 PvGPX4

MLSSSTTRIFIRTTTAIRLASSTSSSLQFLSNSTLFPPLLHASFKPLPSTSTTSFFSSFRTDHTMGTSCFKSVYDFTVKDAKGTEINLGDYKGKVLIIVNVASQCGLTNSNYTELSQLYEKYKQKGLEILAFPCNQFGAQEPGSNEQIQEFVCTRFKAEFPVFDKVDVNGDKADPLYKYLKSSKGGLFGDNIKWNFSKFLVDKEGKVVDRYAPTTSPLSMEKDLLKLLDA

>Phvul.002G322400.1 PvGPX5

MNLLTFWNCISILFLLLAFFIYSHTYPSTPSLMAEESSKSIYDFTVKDIRGNDVSLNDFTGKVILIVNVASQCGLTQTNYKELNVLYDKYKNQGFEILAFPCNQFAGQEPGNNEEIQDVVCTRFKAEFPIFDKVEVNGKNAAPLYKFLKEQKGGIFGDGIKWNFTKFLVNKEGKVVERYAPTTSPLKIEKDIEKLLQS

>Potri.001G105100.1 PtGPX1

QTDRHVRCENTICKSQNSKQVLMIPSHLLLLHLLLSASLASSMATQTSKNPESVHDFTIKDAKENDVDLSIFKGKVLLIVNVASKCGMTNSNYAEMNQLYEKYKDQGLEILAFPCNQFGEEEPGTNDQITDFVCTRFKSEFPIFDKIDVNGENASPLYKFLKLGKWGIFGDDIQWNFAKFLVNKDGQVVDRYYPTTSPLSLEIFHSPDFGCCKLVWIWLLLT

>Potri.003G126100.1 PtGPX2

MLTSRSRILSQKYLNFASLSASFLLSKQSSFNSKQTLLPSLHNSPVSLHSQSIKAGVSRRLLGSVRFNHSMASQSSPQSAHDFTVKDAKGNDVDLSIYKGKVLLIVNVASQCGLTDSNYTELTQLYAKYKDQGLEILAFPCNQFGSQEPGSSEEIVEFACTRFKAEYPIFDKVEVNGNNAAPIYKYLKSSKGGLFGDNIKWNFSKFLVDKEGKVVDRYAPTTSPLSIEKEVKKLLGIA

>Potri.006G265400.1 PtGPX3

MASLPFSCSSVPGLKIHPNPTKMASFVISIKSSLGPSKSAFLQRGFSLQSPNLPGFASKARSFGVFARAATEKSVHDFTVKDINGKDVALSKFKGKALLIVNVASKCGLTSSNYSELTHIYEKYKTQGFEILAFPCNQFGGQEPGSNPEIKQFACTRYKAEFPIFDKVDVNGPSTAPVYQFLKSSAGGFLGDLIKWNFEKFLVDKNGKVVERYQPTTSPFQIEKDIQKLLAA

>Potri.007G126600.1 PtGPX4

MLAFYTMHFTNSISLVFLGFAILALYSYPSLLPSRKMAEESPKSIYDFTVKDIHGNDTSLSEYSGKVLLIVNVASKCGLTHSNYKELNVLYEKYKNQGFEILAFPCNQFAGQEPGSNEEIQDTVCTIFKAEFPIFDKIDVNGKNTAPVYKFLKSEKGGYFGDAIKWNFTKFLVNKEGKVVERYAPTTSPLKIEKDIQNLLGSS

>Potri.014G138800.1 PtGPX5

MGSSPSVPEKSIHEFTVKDNRGQDVNLGIYKGKVLLVVNVASKCGFTDSNYTQLTDLYKNYKDKGLEILAFPCNQFLNQEPGTSEDAQNFACTRYKADYPIFHKVRVNGPNAAPVYKFLKASKPGFLGNRIKWNFTKFLVDKDGHVLGRYSTITAPMAIEADIKKALGEM

>XP_008802381.1 PdGPX1

maaempdsiyditvkdmsgndvslstysgkvllmvnvaskcglthsnykemnalyekykdkgfeilafpcnqfagqepgnneeikevactmfkaefpifdkievngknaaplykflksqkggifgdgikwnftkflvakdgkvmeryapttsplkiekdiqklles

XP_008794200.1 PdGPX2

masnssqatqtvhdltvkdaggndvdlsiykgkvllivnvasqcgltnsnytelstlyerykdkgleilafpcnqfgaqepgtneqildfactrfkaeypifdkvdvnggnaapiykflksrkgslfgsgikwnftkflvdkeghvvdryapttsplsfekkikkllglv

>XP_008790648.1 PdGPX3

maaekpnsiyditvkdmsgsdvslstysgkvllivnvaskcglthsnykemnvlyekykdkgfeilafpcnqfagqepgsneeiqevactrfkaefpifdkievngknaaplykflksqkgglfgdgikwnfskflvakdgkvmeryapttsplkiekdikkllea

>XP_008790151.1 PdGPX4

mssmatastlfsatsaslhgfarpgrtnapalaflhlssklpsgfpktpfrdyrcssqfqknpgfaakplrtpgvayataateksihdftvkdiegkdvslskfkgkvllivnvaskcglttsnytelshiyekyktqgfdilafpcnqfggqepgsnseikqfactrykaefpifdkvdvngpntapvyqflkssaggflgdlvkwnfekflvdkngkvveryppttspfqierdirkllaa

>XP_008775339.1 PdGPX5

mlcsppaalsrfvylrnlshsaslrlskpcsspsqrtlvfapsrissspsrnlrgvgfpfpvssspaasplfcrfasemaskssqatqtvhdftvkdargndvdlsiykgkvllivnvasqcgltnsnytdlttlyekykdkgleilafpcnqfgaqepgtneqilefactrfkaeypifdkvdvngdnaapiykflksskgslfgdsikwnfskflvdkeghvvdryapttsplsiekdvkkllgln

>Medtr1g014210.1 MtGPX1

MVSMASSTTFFTPLHNFNQARTNSIPSISLPFVKSSIPSSKSPFFQHGFSQPTSFDFPKAVLKSRSFSVNARAVTDKSIYDFTVKDIDKKDVPLSKFKGKVLLIVNVASRCGLTSSNYTELSHLYENFKDKGLEVLAFPCNQFGMQEPGSNEEIKKFACTRFKAEFPIFDKVDVNGPFTAPVYQFLKSSSGGFFGDLVKWNFEKFLVDKNGKVVERYPPTTSPFQIEKDIQKLLAA

>Medtr7g094600.1 MtGPX2

MGASHSVSENSIHEFTVKDARGKDVNLSTYKGKVIIVVNVASKCGFTNVNYTQLTELYSRYRDKGLEILAFPCNQFLNQEPGNSLEAEQFACTRFKAEYPIFGKIRVNGPDTAPLYKFLKEKKSGFLGSRIKWNFTKFLVDKEGHVLQRYSPTTSPFSIENDIKKALGET

>Medtr8g098400.2 MtGPX3

MSTEPSNSKDPKSVYDFTLKDGMGNDVDLATYKGKVLLIVNVASKCGMTNSNYVGLNQLYDKYKLKGLEILAFPSNQFGEEEPGTNDQILDFVCTHFKSEFPIFDKIEVNGDNSAPLYKFLKSGKWGIFGDDIQWNFAKFLVDKDGQVVDRYYPTTSPLSLEVHGALLFKKFIFFIYININ

>Medtr8g098410.1 MtGPX4

MLCSTSTTTRIRFISTTKRLLTAPLSSLLRFFSISTTLPNKPIIHKPLFTTLTPSLYFTLRRTDHTMASASNPQSIHDFTVKDAKGNDVNLGDYKGKVLIIVNVASQCGLTNSNYTELSQLYEKYKSKGLEILAFPCNQFGAQEPGSVEEIQNFVCTRFKAEFPVFDKVDVNGATAAPIYKYLKSSKGGLFGDGIKWNFSKFLVDKNGNVVDRYAPTTSPLSIEKDLLKLLDA

>Medtr8g105630.1 MtGPX5

MAENSSKSIYDFTVKDISGNDVSLSQYRGKVLLVVNVASQCGLTQTNYKELNVLYQKYKDQDFEILAFPCNQFRGQEPGSSEEIQNVVCTRFKAEFPVFDKVEVNGKNAEPLYKFLKDQKGGIFGDGIKWNFTKFLVNKEGKVVDRYAPTTAPLKIEKDIEKLLRSS

>Cla011456 ClGPX1

MLREMISTLGYTRVKSYSLSTLLPNGLEILAFPCNQFGDEEPGSNDEIKDFVCSRYKSEFPIFDKIEVNGNNSAPLYKFLKLGKWGIFGDDIQWNFAKFLIDKDGKVVDRYYPTTSPLSIEHDIKKLLGSYEQWSIQV

>Cla011457 ClGPX2

MATPSKTSVHDFTVKDARGNDVDLSIYKGKVLLIVNVASQCGLTNSNYTELSQLYEKYKGHGFEILAFPCNQFGGQEPGTNEEIVQFACTRFKAEYPIFDKVDVNGNNAAPLYKFLKSSKGGLFGDAIKWNFSKFLVDKDGNVVDRYAPTTSPLSIEVNESNSSSQLIL

>Cla021039 ClGPX3

MSFSASFSSPINVCSKTSTAFCYSLASWPSMAANLIPSVKSSLAASKSPFLCHNFPMQSSISRGVFSKVQFSAVSARAATEKSIYDFTVKDIDGKDVSLNKFKGKVLLIVNVASRCGLTTANYSELSHLYEKYKAQGLEILAFPCNQFGGQEPGSNPEIKQFACSRFKAEFPIFDKVDVNGPNTAPVYQFLKSSAGGFLGDLIKWNFEKFLVDKNGKVVERYPPTTSPFQIEKDIQKLVAA

>Cla006080 ClGPX4

MGASQSVPEKSIHEFVVKDARGQDVDLSIYRGKVLLVVNVASKCGYTDSNYTQLTELYSKYKEKGIGSSSSFVQWIGDLGFSMQSVFESGTWIQRGGTGICLYKIQSRVSNIPKDNQRLFLIIQ

>Cla010856 ClGPX5

MAEESSNSIYDFTVKDIRGNDVSLSQYRGKVLLIVNVASECGLTKSNYKELNVLYEKYKNQGFEILAFPCNQFAAQEPGNNEQIQETVCTRFKAEFPIFDKVDVNGKDAAPIYKFLKSQKGGRGLFGDGIKWNFTKFLVNKEGKVVDRYAPTTSPLKIEKDIENLLQSA

>Cla014745 ClGPX6

MGASQSVAEKSIHEFTVKDFKGKDVNLNVYKGKVLLVVNVASKCGFTDSNYSQLTDLYNRYKDQDFEILAFPCNQFLKQEPGTSQDAQEFACTRYKAEYPIFHKVRVNGPDTAPVYKFLKATSNGFLGSRIKWNFTKFLVDKEGVVINRFGPTTSPLAIEDDIKKALGVA

>Brara.B02692.1 BrGPX1

MLRSSFRLLYITRTNLLVRASPSSLSRLSSKFSSAKPLVSSHHQIPLSTTGAKLSRSEHSMAASSEPKSIYDFTVKDAKGNDVDLSTYKGKVLLIVNVASQCGLTNSNYTELAQLYQKYKDHGFEILAFPCNQFGNQEPGSNEEIVQFACTRFKAEYPIFDKVDVNGDSAAPIYKFLKSSKGGLFGDGIKWNFAKFLVDKDGNVVDRYAPTTSPLSIEKDLKKLLGVTA

>Brara.C02198.1 BrGPX2

MPRSRTCVLILLLPLAFVFYLYMSLPSPAIVDQSSYSSIYHISVKDIEGNNVSLSKFTGKVLLIVNVASKCGLTQGNYKELNILYAKYKTKGLEILAFPCNQFGGQEPGSNKEIKENICTTFKGEFPIFDKIEVNGENASPLYKFLKEQKGGLFGDSIKWNFAKFLVDKQGNVVDRYAPTTSPLEIEKDIEKLLASA

>Brara.E00003.1 BrGPX3

MGASVSVPERSVHQFTVKDSSGKDVNLSIYQGKVLLLVNVASKCGFTESNYTQLTELYRKYKDQGFEILAFPCNQFLYQEPGTSQEAHEFACTRFQAEYPVFQKVRVNGQNAAPLYKFLKASKPTFLGSRIKWNFTKFLVSKDGIVIDRYGTMATPLSIEKDIKKALEEA

>Brara.E01208.1 BrGPX4

MADESPKSIYDFTVKDISGNDVSLSQFKGKTLLIVNVASKCGLTDANYKELNVLYEKYKEQGLEILAFPCNQFLGQEPGNNEEIQQTVCTRFKAEFPIFDKVDVNGKNTAPLYKYLKAEKGGLLIDAIKWNFTKFLVSPDGKVSQRYSPRTSPLQFEKDIQALLGQASS

>Brara.G01994.1 BrGPX5

MGGSVSVSEKSIHEFTVKDSSGKEVDLSVYQGKVLLIVNVASKCGFTETNYTQLTELYRKYKDQGLVILAFPCNQFLNQEPGTSQDAHEFACTRFKAEYPVFQKVRVNGQNAAPVYKFLKSKKPSFLGSRIKWNFTKFLVGKDGQVIDRYGPTVPPLSIEKDIKKALGDDGAFPST

>Brara.I01234.1 BrGPX6

MATKDPESVYDISIEDANGNSLELSQYKDKVLLIVNVASKCGMTNSNYTELNEIYNKYKDKGLEILAFPCNQFGEEEPGTTDQITEFVCTKFKSEFPIFNKIEVNGGNASPLYKFLKKGKWGIFGDDIQWNFAKFLVDKNGQAVERYYPTTSPLTLEHDIKKLLNVS

>Brara.I04448.1 BrGPX7

MAFSSSYYSPFSALFDVSKPNPSLNPAAFLVPSLKFSTAISNFANGFSLKSPINPGFLFKSRTFNVQARAAAEKTVHDFTVKDIDGNDVSLNKYKGKVMLIVNVASRCGLTSSNYSELSHLYEKYKSQGFEILAFPCNQFGGQEPGSNPEIKQFACTRFKAEFPIFDKVDVNGPSTAPIYQFLKSNAGGFLGDLIKWNFEKFLIDKKGKVVERYPPTTSPFQIEKDIKKLLAA

>Brara.K00392.1 BrGPX8

MASSSYAPFSAVFSGFAATKPNPPPTCSAFLVPKRRSNSRNLKNGVSLKSWNKHGFQFTSRNLSVYARATEEKTVHDFTVKDISGKDVSLDKFKGKPLLIVNVASKCGLTSSNYTELSQLYDKYRNQGFEILAFPCNQFGGQEPESNPDIKRFVCTRFKAEFPIFDKVDVNGPSTAPIYQFLKSKSGGFLGDLIKWNFEKFLVDKKGNVVQRYPPTTSPLQIEKDIQKLLVA
