# Supplementary material for: Identification of Glutathione Peroxidase Gene Family in Ricinus communis and Functional Characterization of RcGPX4 in Cold Tolerance
Source: Front Plant Sci. 2021 Nov 5;12:707127. doi: 10.3389/fpls.2021.707127 (PMC8602854; doi:10.3389/fpls.2021.707127)
Supplement: Supplementary file 3 [file Table_2.DOCX]

**Supplementary Table 2** Primer sequences for the qRT-PCR assay in this study

| **Gene Name** | **5’ primer (5’→3’)** | **3’ primer (5’→3’)** |
| --- | --- | --- |
| *RcGPX1* | GGCTTCTCCATCAGTTCCAG | TCCAGGCTCTTGTTTCAGG |
| *RcGPX2* | TCTAGTAAACAAGGAGGGCAAGG | TGGAAAGCAAACCCACAACAC |
| *RcGPX3* | ATTGGCAACTTCTTCTGGG | TGCTGAGCGGTACGTCTTT |
| *RcGPX4* | CAAAGCAAACCCTTCTGC | AACTATCAAGAGAGCCTTCCC |
| *RcGPX5* | ATACTACCCAACCACTTCTCCT | ATGTCCCATACAATCATCACG |
| *RcADP* | GAGAGATGCTGTGCTGCTTG | TGAAGGCCGAGCTTATCAGT |
| *RcEF1β* | GCTCGAGGAAGCAGTTAGGA | TTCCATATCCAACTGGCACA |
| *AtMAPK3* | GCCCTTAGCTAAACTTTTCTC | CGTGCAATTTAGCAAGGTACT |
| *AtCBF1* | GCATGTCTCAACTTCGCTGA | ATCGTCTCCTCCATGTCCAG |
| *AtCBF2* | TGACGTGTCCTTATGGAGCTA | CTGCACTCAAAAACATTTGCA |
| *AtICE1* | AGCTCCGTTGGAGTTGGAAG | GCTGCACGTTTCTGGAACAG |
| *AtCOR47* | CAGTGTCGGAGAGTGTGGTG | ACAGCTGGTGAATCCTCTGC |
| *AtKIN1* | ACCAACAAGAATGCCTTCCA | CCGCATCCGATACACTCTTT |
| *AtRD29A* | GCCGAGAAACTTCAGATTGG | CCATTCCTCCTCCTCCTTTC |
| *AtABI1* | AGCTGCTGATATAGTCGTCGTTGATA | GAGGATCAAACCGACCATCTAACA |
| *AtABI2* | GTTCTTGTTCTGGCGACGGAGC | CCATTAGTGACTCGACCATCAAG |
| *AtRAB18* | GCAGTATGACGAGTACGGAAATCC | CCTTGTCCATCATCCGAGCTAGA |
| *AtACTIN2/8* | GGTAACATTGTGCTCAGTGGTGG | AACGACCTTAATCTTCATGCTGC |
| *AtTubulin* | CCAACAACGTGAAATCGACAG | TCTTGGTATTGCTGGTACTCT |
