# Supplementary material for: Identification of Glutathione Peroxidase Gene Family in Ricinus communis and Functional Characterization of RcGPX4 in Cold Tolerance
Source: Front Plant Sci. 2021 Nov 5;12:707127. doi: 10.3389/fpls.2021.707127 (PMC8602854; doi:10.3389/fpls.2021.707127)
Supplement: Supplementary file 4 [file Table_3.DOCX]

**Supplementary Table 3** Conserved motifs in the deduced amino acid sequences of GPXs from castor bean, rice, and *Arabidopsis*

| Motif | Width | E-value | Protein domain^a^ | Multilevel Consensus Sequences |
| --- | --- | --- | --- | --- |
| 1 | 50 | 1.0e-579 | GSHPx  (PF00255) | 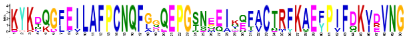 |
| 2 | 50 | 9.4e-484 | GSHPx  (PF00255) | 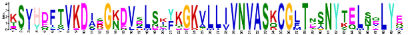 |
| 3 | 50 | 3.3e-483 | Not found | 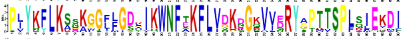 |
| 4 | 50 | 5.7e-004 | Not found | 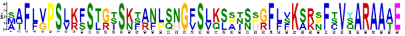 |
| 5 | 6 | 2.6e-003 | Not found | 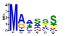 |

^a^ The GPX domain was retrieved from Pfam database.
