# Supplementary material for: Identification of Glutathione Peroxidase Gene Family in Ricinus communis and Functional Characterization of RcGPX4 in Cold Tolerance
Source: Front Plant Sci. 2021 Nov 5;12:707127. doi: 10.3389/fpls.2021.707127 (PMC8602854; doi:10.3389/fpls.2021.707127)
Supplement: Supplementary file 5 [file Table_4.DOCX]

**Supplementary Table 4** List of subcellular localization analyses from 13 plant species including 75 GPX homologs

| **Protein ID** | **Gene Name** | **Localization**  **CELLO^a^** | **Localization**  **WoLF PSORT** | **TargetP** |
| --- | --- | --- | --- | --- |
| **Chloroplast and Mitochondria related proteins** | | | | |
| AT2G25080 | AtGPX1 | Chlo/Mito | Chlo | Chlo |
| Thhalv10000311m | TsGPX1 | Chlo/Mito | Chlo | Chlo |
| Brara.I04448.1 | BrGPX7 | Mito/Chlo | Chlo | Chlo |
| AT4G31870 | AtGPX6 | Chlo | Chlo | Chlo |
| Thhalv10026852m | TsGPX7 | Chlo/Mito | Chlo | Chlo |
| Brara.K00392.1 | BrGPX8 | Mito | Chlo | Chlo |
| GSVIVG01035981001 | VvGPX5 | Chlo/Mito | Chlo | Chlo |
| Potri.006G265400 | PtGPX3 | Chlo/Mito | Chlo | Chlo |
| Phvul.001G041100 | PvGPX1 | Mito | Chlo | Mito |
| Medtr1g014210 | MtGPX1 | Mito/Chlo | Chlo | Chlo |
| Solyc08g006720.2 | SlGPX2 | Chlo | Chlo | Chlo |
| Csa4M651840.1 | CsGPX2 | Chlo | Chlo | Chlo |
| Cla021039 | ClGPX3 | Chlo | Chlo | Chlo |
| XP_008790151 | PdGPX4 | Mito/Chlo | Chlo | Chlo |
| 29848.m004526 | RcGPX3 | Chlo | Chlo | Chlo |
| LOC_Os06g08670 | OsGPX4 | Mito/Chlo | Chlo | Chlo |
| LOC_Os02g44500 | OsGPX1 | Chlo | Chlo | Mito |
| LOC_Os04g46960 | OsGPX3 | Extr | Chlo | Other |
| **Cytoplasm related proteins** | | | | |
| AT1G63460 | AtGPX7 | Cyto | Cyto | Other |
| Brara.I01234.1 | BrGPX6 | Cyto | Cyto | Other |
| Thhalv10023725m | TsGPX6 | Cyto | Cyto | Other |
| Csa5M154190.1 | CsGPX3 | Cyto | Cyto | Other |
| Cla011456 | ClGPX1 | Cyto | Chlo | Mito |
| 30190.m011205 | RcGPX5 | Cyto | Chlo | Other |
| Potri.001G105100 | PtGPX1 | Extra | Cyto | Signal peptide |
| Medtr8g098400 | MtGPX3 | Cyto | Cyto | Other |
| GSVIVG01019765001 | VvGPX3 | Cyto | Cyto | Other |
| Solyc12g056240.1 | SlGPX5 | Cyto | Cyto | Other |
| Phvul.002G157200 | PvGPX3 | Cyto | Chlo | Other |
| **Cytoplasm, Extracellular, Plasma membrane related proteins** | | | | |
| AT2G31570 | AtGPX2 | Cyto | Cyto | Other |
| Brara.E01208.1 | BrGPX4 | Cyto | Cyto | Other |
| Thhalv10017319m | TsGPX5 | Cyto | Cyto | Other |
| Cla010856 | ClGPX5 | Cyto | Cyto | Other |
| 29657.m000470 | RcGPX2 | Cyto | Cyto | Other |
| GSVIVG01010737001 | VvGPX1 | Cyto | Cyto | Other |
| XP_008802381 | PdGPX1 | Cyto | Cyto | Other |
| LOC_Os11g18170 | OsGPX5 | Chlo/Extr | Chlo | Signal peptide |
| Solyc06g073460.2 | SlGPX1 | Cyto | Chlo | Other |
| Medtr8g105630 | MtGPX5 | Cyto | Chlo | Other |
| Phvul.002G322400 | PvGPX5 | Extr | Extr | Signal peptide |
| Potri.007G126600 | PtGPX4 | Extr | Extr | Signal peptide |
| Csa4M013040.1 | CsGPX1 | Cyto | Chlo | Other |
| Brara.C02198.1 | BrGPX2 | Extra/Plas | Extra | Signal peptide |
| Thhalv10001645m | TsGPX2 | Extra/Plas | Vacu | Signal peptide |
| AT2G43350 | AtGPX3 | Mito/Plas | Chlo | Other |
| XP_008790648 | PdGPX3 | Cyto | Nucl | Other |
| **Cytoplasm, Chloroplast, and Mitochondria related proteins** | | | | |
| Csa5M154200.1 | CsGPX4 | Cyto | Chlo | Other |
| Cla011457 | ClGPX2 | Cyto | Chlo | Other |
| Thhalv10028932m | TsGPX8 | Chlo/Mito | Chlo | Mito |
| Brara.B02692.1 | BrGPX1 | Mito | Chlo | Mito |
| XP_008794200 | PdGPX2 | Cyto | Chlo | Other |
| XP_008775339 | PdGPX5 | Mito/Chlo | Chlo | Chlo |
| 30190.m011204 | RcGPX4 | Chlo/Mito | Chlo | Mito |
| Phvul.002G288700 | PvGPX4 | Chlo/Mito | Chlo | Mito |
| Medtr8g098410 | MtGPX4 | Mito | Mito | Mito |
| GSVIVG01019766001 | VvGPX4 | Cyto | Chlo | Other |
| Potri.003G126100 | PtGPX2 | Mito/Chlo | Mito | Mito |
| Solyc08g080940.2 | SlGPX3 | Mito | Chlo | Mito |
| **Cytoplasm, Extracellular, Nucleus related proteins** | | | | |
| LOC_Os03g24380 | OsGPX2 | Cyto | Cyto | Other |
| Solyc09g064850.2 | SlGPX4 | Mito/Extr | Chlo | Other |
| 28153.m000283 | RcGPX1 | Cyto/Extra | Chlo | Other |
| Potri.014G138800 | PtGPX5 | Cyto/Extr | Chlo/Cyto | Other |
| Cla006080 | ClGPX4 | Extra/Nuc | Chlo | Other |
| Csa6M408810.1 | CsGPX5 | Cyto/Extr | Cyto | Other |
| Medtr7g094600 | MtGPX2 | Cyto/Mito | Nucl | Other |
| Phvul.001G149000 | PvGPX2 | Cyto/Nucl | Nucl | Other |
| GSVIVG01011101001 | VvGPX2 | Cyto | Mito | Other |
| Cla014745 | ClGPX6 | Cyto/Extra | Chlo | Other |
| Csa7M392410.1 | CsGPX6 | Cyto | Chlo | Other |
| Thhalv10001660m | TsGPX3 | Extra | Chlo | Other |
| Brara.E00003.1 | BrGPX3 | Extra/Cyto | Chlo | Other |
| AT2G48150 | AtGPX4 | Cyto | Chlo | Other |
| AT3G63080 | AtGPX5 | Extr/Chlo/Nucl | Chlo | Other |
| Brara.G01994.1 | BrGPX5 | Cyto/Extra/Nucl | Chlo | Other |
| Thhalv10006271m | TsGPX4 | Extra | Chlo | Other |

^a^ Cyto, Cytoplasm; Chlo, Chloroplast; Mito, Mitochondria; Nucl, Nucleus; Extr, Extracellular; Plas, Plasma membrane.
