# Supplementary material for: Identification of Glutathione Peroxidase Gene Family in Ricinus communis and Functional Characterization of RcGPX4 in Cold Tolerance
Source: Front Plant Sci. 2021 Nov 5;12:707127. doi: 10.3389/fpls.2021.707127 (PMC8602854; doi:10.3389/fpls.2021.707127)
Supplement: Supplementary file 6 [file Table_5.DOCX]

**Supplementary Table 5** *Cis*-regulatory elements identified in all *RcGPX* genes

| Gene | Elements for light response | Elements for hormone response | Elements stress response | Elements for tissue specific expression |
| --- | --- | --- | --- | --- |
| *RcGPX-1* | ACA-motif | ABRE | ARE | CAT-box |
|  | AE-box (2)* |  | MYB (3)* |  |
|  | Box 4 (2)* | P-box | MYC (4)* |  |
|  | G-box |  | MBS (1)* |  |
|  | GA-motif |  |  |  |
|  | GT1-motif (2)* |  |  |  |
|  | MRE |  |  |  |
|  | TCT-motif |  |  |  |
| *RcGPX-2* | LAMP-element | ABRE (2) | MBS |  |
|  | TCT-motif | CGTCA-motif | MYB |  |
|  | sbp-CMA1c | GARE-motif | MYC (2) |  |
|  | Box 4 (9) | TCA-element (2) |  |  |
|  |  | TGACG-motif |  |  |
|  | G-Box (2) |  |  |  |
| *RcGPX-3* | AE-box |  | ARE (4) | CAT-box |
|  | GATA-motif |  | MBS |  |
|  | GT1-motif |  | MYB (2) |  |
|  | I-box |  | MYC (2) |  |
| *RcGPX-4* | LAMP-element | TGA-element | ABRE (3) | CAT-box |
|  | chs-CMA1a |  | ARE (3) |  |
|  |  |  | LTR |  |
|  | Box 4 (2) |  | MBS |  |
|  | G-Box (3) |  | MYB (2) |  |
|  | GT1-motif (2) |  |  |  |
| *RcGPX-5* | 3-AF1 binding site | ABRE (4) | MYB (2) | O_2_-site |
|  | AE-box | TCA-element | MYC(2) |  |
|  | LAMP-element |  | TC-rich repeats |  |
|  | Box 4 (2) |  | WUN-motif |  |
|  | GT1-motif |  |  |  |
|  | G-Box (4) |  |  |  |
|  | GATA-motif |  |  |  |
|  | MRE |  |  |  |

* represents the number of the elements
